# An *miR164*-resistant mutation in the transcription factor gene *CpCUC2B* enhances carpel arrest and ectopic boundary specification in *Cucurbita pepo* flower development

**DOI:** 10.1093/jxb/erad486

**Published:** 2023-12-08

**Authors:** María Segura, Alicia García, Germán Gamarra, Álvaro Benítez, Jessica Iglesias-Moya, Cecilia Martínez, Manuel Jamilena

**Affiliations:** Department of Biology and Geology. Agri-food Campus of International Excellence (CeiA3) and Research Center CIAIMBITAL, University of Almería, 04120 Almería, Spain; Department of Biology and Geology. Agri-food Campus of International Excellence (CeiA3) and Research Center CIAIMBITAL, University of Almería, 04120 Almería, Spain; Department of Biology and Geology. Agri-food Campus of International Excellence (CeiA3) and Research Center CIAIMBITAL, University of Almería, 04120 Almería, Spain; Department of Biology and Geology. Agri-food Campus of International Excellence (CeiA3) and Research Center CIAIMBITAL, University of Almería, 04120 Almería, Spain; Department of Biology and Geology. Agri-food Campus of International Excellence (CeiA3) and Research Center CIAIMBITAL, University of Almería, 04120 Almería, Spain; Department of Biology and Geology. Agri-food Campus of International Excellence (CeiA3) and Research Center CIAIMBITAL, University of Almería, 04120 Almería, Spain; Department of Biology and Geology. Agri-food Campus of International Excellence (CeiA3) and Research Center CIAIMBITAL, University of Almería, 04120 Almería, Spain; University College Dublin, Ireland

**Keywords:** Boundary specification, *CUC2*, *Cucurbita pepo*, ethylene, female flowering, flower meristem

## Abstract

The sex determination process in cucurbits involves the control of stamen or carpel development during the specification of male or female flowers from a bisexual floral meristem, a function coordinated by ethylene. A gain-of-function mutation in the *miR164*-binding site of *CpCUC2B*, ortholog of the Arabidopsis transcription factor gene *CUC2*, not only produced ectopic floral meristems and organs, but also suppressed the development of carpels and promoted the development of stamens. The *cuc2b* mutation induced the transcription of *CpCUC2B* in the apical shoots of plants after female flowering but repressed other *CUC* genes regulated by *miR164*, suggesting a conserved functional redundancy of these genes in the development of squash flowers. The synergistic androecious phenotype of the double mutant between *cuc2b* and *etr2b*, an ethylene-insensitive mutation that enhances the production of male flowers, demonstrated that *CpCUC2B* arrests the development of carpels independently of ethylene and *CpWIP1B*. The transcriptional regulation of *CpCUC1*, *CpCUC2*, and ethylene genes in *cuc2b* and ethylene mutants also confirms this conclusion. However, the epistasis of *cuc2b* over *aco1a*, a mutation that suppresses stamen arrest in female flowers, and the down-regulation of *CpACS27A* in *cuc2b* female apical shoots, indicated that *CpCUC2B* promotes stamen development by suppressing the late ethylene production.

## Teaser

The *CpCUC2B* boundary gene plays an essential role in squash sex determination. The gene prevents carpel formation, independently of ethylene, and promotes stamen development by inhibiting the ethylene biosynthesis gene *CpACS27A*.

## Introduction

The zucchini morphotype of *Cucurbita pepo* is one of the most economically significant cucurbit crops. The species is monoecious, carrying unisexual male and female flowers on the same individual plant, but the number of female and male flowers per plant varies between the different cultivars. Unisexual flowers are individually developed at each node in the main and secondary shoots, with the plant exhibiting three sequential sexual developmental phases: an initial male phase; a mixed phase characterized by alternating production of female and male flowers; and a predominantly or exclusively female phase (Peñaranda *et al.*, 2007; Manzano *et al.*, 2010, 2013). These phases are regulated by a combination of environmental, hormonal, and genetic factors (Martínez and Jamilena, 2021).

Ethylene has been established as the primary regulator of sex determination in cucurbits (Manzano *et al*., 2010; Pannell, 2017). Elevated levels of this hormone promote female flower development and facilitate the maturation of floral organs (Manzano *et al.*, 2013), whereas suppression of ethylene production or impairment of its perception leads to the conversion of females into male or bisexual flowers, changing monoecy into androecy or andromonoecy (Martínez *et al.*, 2014; García *et al.*, 2020a, b). Several genes associated with this developmental process have been identified in cucurbits, including *ACS* and *ACO* genes, such as *CsACS2* in cucumber, *CmACS7* in melon, *CitACS4* in watermelon, and *CpACS27A* and *CpACO1* in zucchini. Mutations in these genes promote the conversion of female into bisexual or hermaphrodite flowers, indicating their key role in stamen arrest during development of female flowers (Boualem *et al.*, 2008, 2009; Martínez *et al.*, 2014; Manzano *et al.*, 2016; Cebrián *et al.*, 2022). In contrast, loss-of-function mutation in other ethylene biosynthesis genes, including cucumber and melon *ACS11* (Boualem *et al*., 2015) and cucumber *CsACO2* (Chen *et al.*, 2016), converts female into male flowers and thus monoecy into androecy, indicating that they function as promoters of carpel development at early stages of flower development (Martínez and Jamilena, 2021). The sex phenotypes of four ethylene-insensitive mutants in zucchini have also demonstrated that the different ethylene receptors cooperate in the female flower bud to promote the development of carpels and the arrest of stamens during female flower development. Single and double ethylene-insensitive mutants are, in fact, andromonoecious or androecious depending on the dosage and strength of mutant alleles in each genotype (García *et al.*, 2020a, b).

In addition to ethylene biosynthesis and perception genes, various transcription factor genes have been identified in cucurbits that control sex determination. The ethylene-responsive transcription factor genes *ERF110* and *ERF31* not only show an ethylene-sensitive expression but also regulate the production of ethylene by transcriptional activation of the ethylene biosynthesis genes *ACS11* and *ACS2*, respectively (Pan *et al.*, 2018; Tao *et al.*, 2018). The *WIP1* gene of both cucumber and melon encodes a zinc finger transcription factor with a key role in carpel abortion during the development of male flowers and is transcriptionally regulated by *ACS11* and *ACO2* (Boualem *et al*., 2015; Chen *et al.*, 2016). Mutations in *WIP1* lead to ginoecy in melon, cucumber, and watermelon (Martin *et al.*, 2009; Hu *et al.*, 2017; Zhang *et al.*, 2020). The WIP1 transcription factor regulates the development of male flowers by repressing the transcription of the carpel identity gene *CRABS CLAW* (*CRC*) through a TOPLESS-mediated histone deacetylation mechanism, and therefore *CRC* mutants are androecious (Zhang *et al.*, 2022).

This study introduces a new player in the sex determination gene network of the cucurbits, the *C. pepo* transcription factor gene *CUP-SHAPED COTYLEDON 2B* (*CpCUC2B*). We show that an *miR164*-resistant mutation in the *CpCUC2B* boundary specification gene of zucchini not only increased the number of floral meristems, the number of floral organs, and the serration of leaves, but also promoted the conversion of female into male flowers, leading to a nearly androecious phenotype. Therefore, the gene mediates the arrest of carpels during the development of male flowers.

## Materials and methods

### Plant material for mutant isolation

An ethyl methanesulfonate (EMS) mutant collection composed of 3751 families of *C. pepo* was generated at the University of Almeria in the zucchini genetic background MU-CU-16 (García *et al.*, 2018). A high-throughput screening was performed looking for alterations in vegetative and reproductive development in 123 M_2_ families, grown to maturity under standard greenhouse conditions during the spring–summer 2020 season in Almeria, Spain. The phenotypic analysis resulted in the identification of a mutant line (*cuc2b*) with a reduced proportion of female flowers, as well as alterations in the development of the floral organs.

The *cuc2b* locus was mapped by crossing mutant M_2_ plants with the scallop line UPV-196. The F_1_ plants were then self-pollinated and the resulting F_2_ segregating population was used to perform bulk segregant analysis sequencing (BSA-seq). In parallel, mutant M_2_ plants were crossed twice with MU-CU-16 to generate the segregating populations BC1S1 and BC2S1 in the MU-CU-16 background. These segregating plants were evaluated to validate the causal mutation of the mutant phenotype.

### Phenotypic evaluation of the *cuc2b* mutant line

More than 600 BC1S1 and BC2S1 plants were phenotyped for sex expression in the autumn–winter 2021 season and in the spring–summer 2022 season. The sex phenotype of each plant was determined by scoring the presence of pistillate or staminate flowers at the first 60 nodes of the plants. This evaluation led to the classification of plants into three groups: wild type (wt/wt), characterized by a proportion of female flowers comparable with the genetic background (30%); mutants (*cuc2b/cuc2b*) with a reduced percentage of female flowers and alterations in the development of floral organ development; and heterozygotes (wt/*cuc2b*), which show an intermediate phenotype of sex expression.

These plants were also analyzed for the presence of alterations in vegetative and reproductive organs: number of flowers per node, number of floral organs in male and female flowers, as well as alterations in leaf morphology.

### Identification of the *cuc2b* mutation by BSA-seq coupled with whole-genome sequencing

To identify the causal mutation of the *cuc2b* phenotype, wild-type (WT) and mutant plants derived from F_2_ segregating populations were subjected to whole-genome sequencing (WGS). Genomic DNA from 15 WT and 15 *cuc2b* plants was extracted from young leaves using the cetyltrimethylammonium bromide (CTAB) method (Doyle and Doyle, 1987). The concentration of all samples was adjusted for an equal representation of each plant DNA. The WT DNA samples were pooled to create the WT-bulk, and the mutant DNA samples were pooled to create the MUT-bulk. DNA was also sequenced from 10 plants from the scallop line UPV-196.

DNA from each bulk was fragmented to an average size of 350 bp and was end-repaired, A-tailed, and ligated with Illumina adapters. The Illumina NovaSeq 6000 platform was used to perform genome DNA sequencing by Novogene Co., Ltd, generating 150 bp paired-end reads. Sequencing data were aligned with the reference *C. pepo* genome v4.1 using BWA software (Li and Durbin, 2009) (parameters: mem -t 4 -k 32 -M), and single nucleotide polymorphism (SNP) variations were detected using SAMtools (Li, 2011) and BCFtools (Danecek *et al.*, 2021) with the following parameter: mpileup -m 2 -F 0.002'. The resulting SNPs were annotated with the ANNOVAR software (Wang *et al.*, 2010).

The resulting VCF file was used for quantitative trait locus sequencing (QTL-seq) analysis using the QTLseqr package of R (Mansfeld and Grumet, 2018). The SNPs from the two bulks present in the file were filtered using the ‘filterSNPs’ function with the following parameters: total depth between 30 and 200, reference allele frequency 0.3, and genotype quality >50. To detect putative QTLs, the SNP index and ΔSNP index were obtained using the function ‘runQTLseqAnalysis’ (Takagi *et al.*, 2013). Identification of candidate QTL regions was performed using a 1 Mb sliding window, and the confidence intervals (90, 95, and 99%) for the ΔSNP indices were determined using 10 000 simulations for each bulk.

To establish candidate mutations, the SNPs present in the putative QTL were filtered according to the following parameters: alternative allele frequency (AF) in the WT-bulk <0.35, AF>0.65 in the mutant-bulk, genotype quality ≥30, and read depth ≥7. Common variants between this and other mutant lines already sequenced in the laboratory were filtered out, as they are variants that were fixed in the genetic background line MU-CU-16 after *de novo* sequencing and assembly of the reference genome. Those variants that were specific for the scallop line UPV-196 were also discarded. Positions with canonical EMS changes (G>A or C>T transitions) were selected as candidate mutations and used to conduct a fine mapping study to identify the causal mutation of the phenotype.

### Validation of the *cuc2b* mutation by high-throughput genotyping of individual segregating plants

A total of 356 BC1S1 and 280 BC2S1 plants in the genetic background MU-CU-16 were subjected to high-throughput genotyping using competitive allele-specific PCR (KASP) technology according to the manufacturer’s instructions. The primers for the putative SNPs were synthesized by LGC Genomics®, and the KASP assay was performed on the CFX96 Touch real-time PCR detection system (Bio-Rad®) using the LGC KASP genotyping protocol. PCR cycling conditions were analyzed using CFX Maestro™ software (Bio-Rad®) to determine the genotype of each individual plant for the SNP candidates analyzed.

### Bioinformatic analysis: phylogeny, protein stability, and regulatory networks.

Homologous protein sequences and putative *miR164*-binding sites were identified using the NCBI BLAST tool (https://blast.ncbi.nlm.nih.gov). Multiple sequence alignments of CUC2 transcription factors from other species and *miR164*-binding sites were performed with Clustal Omega (Sievers *et al.*, 2011). The secondary structure of *Pri-miR164* was predicted by miRNA-fold (Tav *et al.*, 2016) and the graphic representation was performed using the FORNA online tool (Kerpedjiev *et al.*, 2015).

The phylogenetic relationship of CpCUC2 with other homologous proteins (Supplementary Table S1) was studied using MEGA X software (Kumar *et al.*, 2018) with MUSCLE alignment (Edgar, 2004) and the Maximum Likelihood method based on the Poisson correction mode (Zuckerkandl and Pauling, 1965), with 2000 bootstrap replicates. The analysis of conserved motifs was done with the MEME tool (https://meme-suite.org/meme/tools/meme; Bailey *et al.*, 2015) and motif identification in the PFAM database (Mistry *et al.*, 2021). The gene structure was determined using the GSDS 2 tool (Hu *et al.*, 2015). *Cis*-regulatory elements were analyzed on the PlantCare website (Rombauts *et al.*, 1999). Schematic representations were performed with TBtools (Chen *et al.*, 2020).

The regulatory network of *CpCUC* genes was inferred using the ARACNE algorithm (Margolin *et al.*, 2006) and visualized using Cytoscape software (Shannon *et al.*, 2003). The inputs for the software were an expression matrix derived from the expression data of different plant organs and the *C. pepo* transcription factors of the PlantTFDB (Jin *et al.*, 2017). Minimum mutual information (MI), the value used to measure the dependence between two random variables, was obtained, and interactions with *P*-values <1e^–4^ were selected after 100 bootstrap iterations.

### Gene expression analysis

Three approaches were followed to perform gene expression analysis: (i) RNA-seq of samples from different plant organs of the genetic background MU-CU-16; (ii) RNA-seq of samples from different organs of the WT and ethylene-insensitive mutants *etr2b*, *etr1b*, and *etr1a-1* (García *et al.*, 2020a, b); and (iii) quantitative real-time PCR (qRT-PCR) from apical shoots of WT, *cuc2b* plants, and the ethylene-deficient and insensitive mutants *aco1a* and *etr2b* (García *et al.*, 2020b; Cebrián *et al.*, 2022), respectively.

The following plant organs were analyzed by RNA-seq in MU-CU-16: apical shoots of plants in the developmental stages of three nodes (male apical shoots, before female flowering) and 12 nodes (female apical shoots, after female flowering), male and female flowers with a corolla length of 30 mm, leaves of 10 mm, and roots from seedlings 17–21 d after germination. For each sample, three biological replicates have been taken from at least three plants per replicate. For RNA isolation, the Omega Biotek EZNA® plant RNA kit (R6827-01) was used following the manufacturer’s protocol. The RNA was then eluted in nuclease-free water and immediately prepared for sequencing. Samples were sequenced by BGI Genomics using the DNBseq platform, generating 150 bp paired-end reads and 6 Gb of raw data per sample.

The transcriptomes of the WT and ethylene mutants *etr1b* and *etr1a-1* (García *et al.*, 2020a) were analyzed by RNA-seq in apical shoots of plants with either three or 12 nodes. Library construction and sequencing of ethylene perception mutant samples were performed by Novogene. The sequencing platform used was the Illumina NovaSeq 6000 Sequencing System, generating 150 bp paired-end reads and obtaining 6 Gb of raw data per sample. Fragments per kilobase of transcript per million mapped reads (FPKM) values were obtained for the analysis of results using the BALLGOWN package in R (Frazee *et al.*, 2014, Preprint), and these data were used to create heatmaps with TBtools.

The analysis of gene expression by qRT-PCR was carried out in samples of WT and *cuc2b*, *aco1a* (Cebrián *et al.*, 2022), and *etr2b* (García *et al.*, 2020b) plants grown in a greenhouse during the spring–summer season. Apical shoots from adult plants were collected in triplicate for each genotype, sampling at least five plants per replicate. Total RNA was isolated with the Omega Biotek EZNA® plant RNA Kit (R6827-01). RNA was converted to cDNA with the ADNc RevertAidTM kit (Thermo Fisher Scientific^®^). qPCR was carried out in a 10 μl total volume with 1× Top Green qPCR Super Mix (Bio-Rad®) on the thermocycler of the CFX-96 Touch Real-Time PCR Detection System (Bio-Rad^®^). Gene expression values were calculated using the 2^–ΔΔCT^ method (Livak and Schmittgen, 2001). Two constitutive genes were used as internal reference, *CpEF1α* and *CpACT*. Primers for gene expression analysis are shown in Supplementary Table S2. To validate the *miR164*-binding and cutting site of *CpCUC2B*, two pairs of primers were used for qPCR, one designed in the 3' region of the transcript, and the other flanking the predicted binding site of *miR164* (Supplementary Table S2).

### Statistical analysis

Data were subjected to an ANOVA using the statistical software Statgraphic Centurion XVIII. Differences between samples were separated by least significant difference at a significance level of *P*≤0.05.

## Results

### The mutant *cuc2b* shows a nearly androecious sex phenotype

**Fig. 1.**
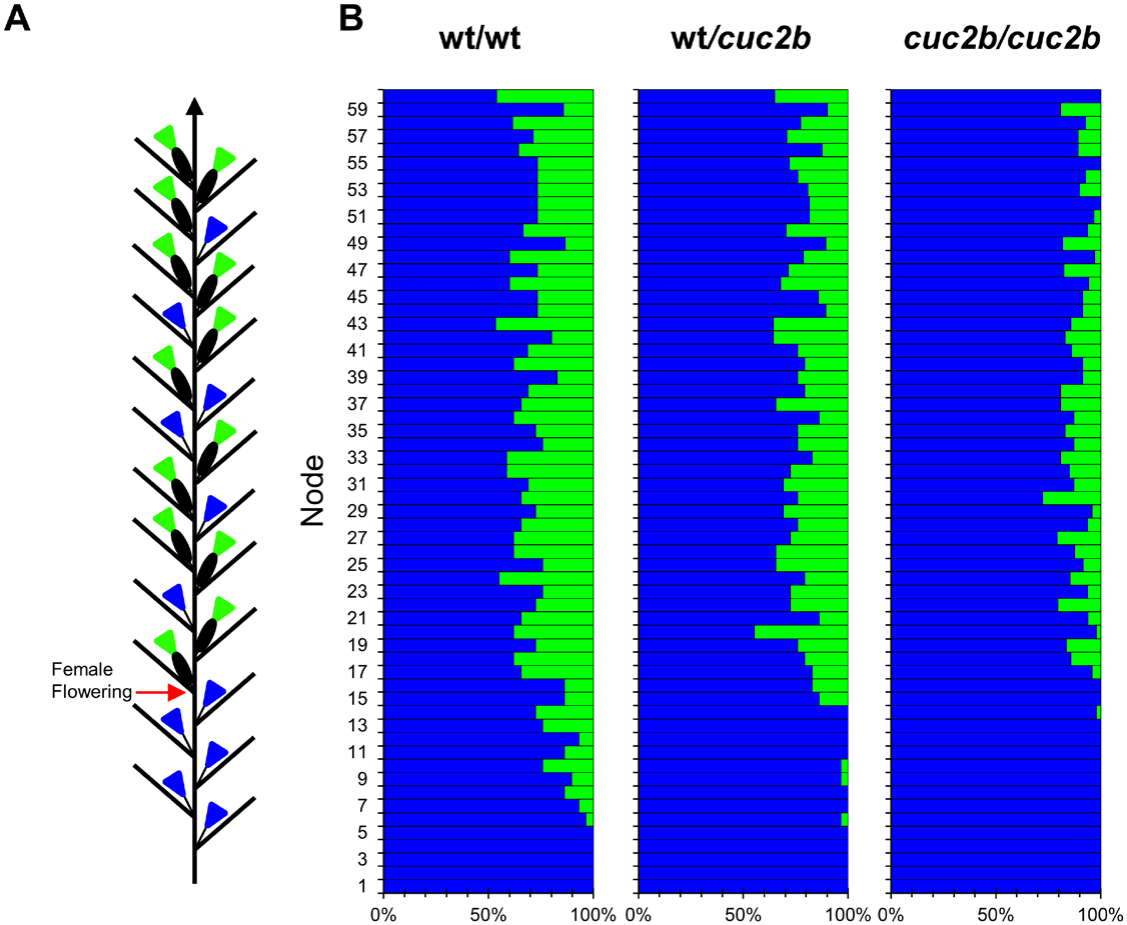
Sex phenotypes of the WT and *cuc2b* mutant. (A) Schematic representation of the distribution of males and females in the monoecious squash plant. Blue=male flower; green=female flower. The red arrow indicates the female flowering transition (FFT), the node at which the first female flower is developed. (B) Distribution of male and female flowers in the first 60 nodes of the wt/wt, wt/*cuc2b*, and *cuc2b/cuc2b* mutant plants (total plants=636). In each node, blue and green bars represent the percentage of male and female flowers of the total plants analyzed, respectively.

The sex phenotype of the monoecious species *C. pepo* is characterized by two different developmental phases in the main shoot, a first male phase in which plants produce only male flowers, and a second female phase in which plants alternate the production of male and female flowers (Fig. 1A). The transition between the two phases, that is, the node at which the plants start to produce female flowers, is called female flowering transition.

The phenotypic screening of an EMS mutant collection of *C. pepo* allowed the identification of a novel mutant called *cuc2b* which is altered in the female flowering transition and the determination of sex. For phenotyping analysis, the mutation was introgressed in the genetic background of MU-CU-16 by two successive backcrosses, and sex phenotyping was performed in 185 plants from the BC2S1 segregating population. In homozygous wt/wt plants, the female flowering transition occurred around node 11, and plants produced an average of 23.7% of female flowers per plant (Table 1; Fig. 1B). Mutant plants showed a very late female flowering transition and a nearly androecious phenotype, with a very reduced number of female flowers per plant (Table 1). Homozygous *cuc2b/cuc2b* plants started female flowering around node 30 and produced an average of only 7.6% female flowers per plant (Table 1). Heterozygous wt/*cuc2b* plants showed an intermediate sex phenotype (Table 1), indicating that the mutation is semi-dominant.

**Table 1.** Sex expression of WT and *cuc2b* mutant plants

Genotype	Female flowering transition	Female flowers per plant (%)
**wt/wt**	11.8 ± 0.6 a	23.7 ± 1.2 a
**wt/*cuc2b***	15.8 ± 0.4 b	19.0 ± 0.7 b
***cuc2b*/*cuc2b***	30.4 ± 2.7 c	7.6 ± 1.1 c

Female flowering transition indicates the node at which the first female flower emerges. Different letters in the same column indicate significant differences between phenotypes (ANOVA, *P*≤0.05).

### The *cuc2b* flowers are impaired in the specification of the floral meristem and the separation of floral organs

**Fig. 2.**
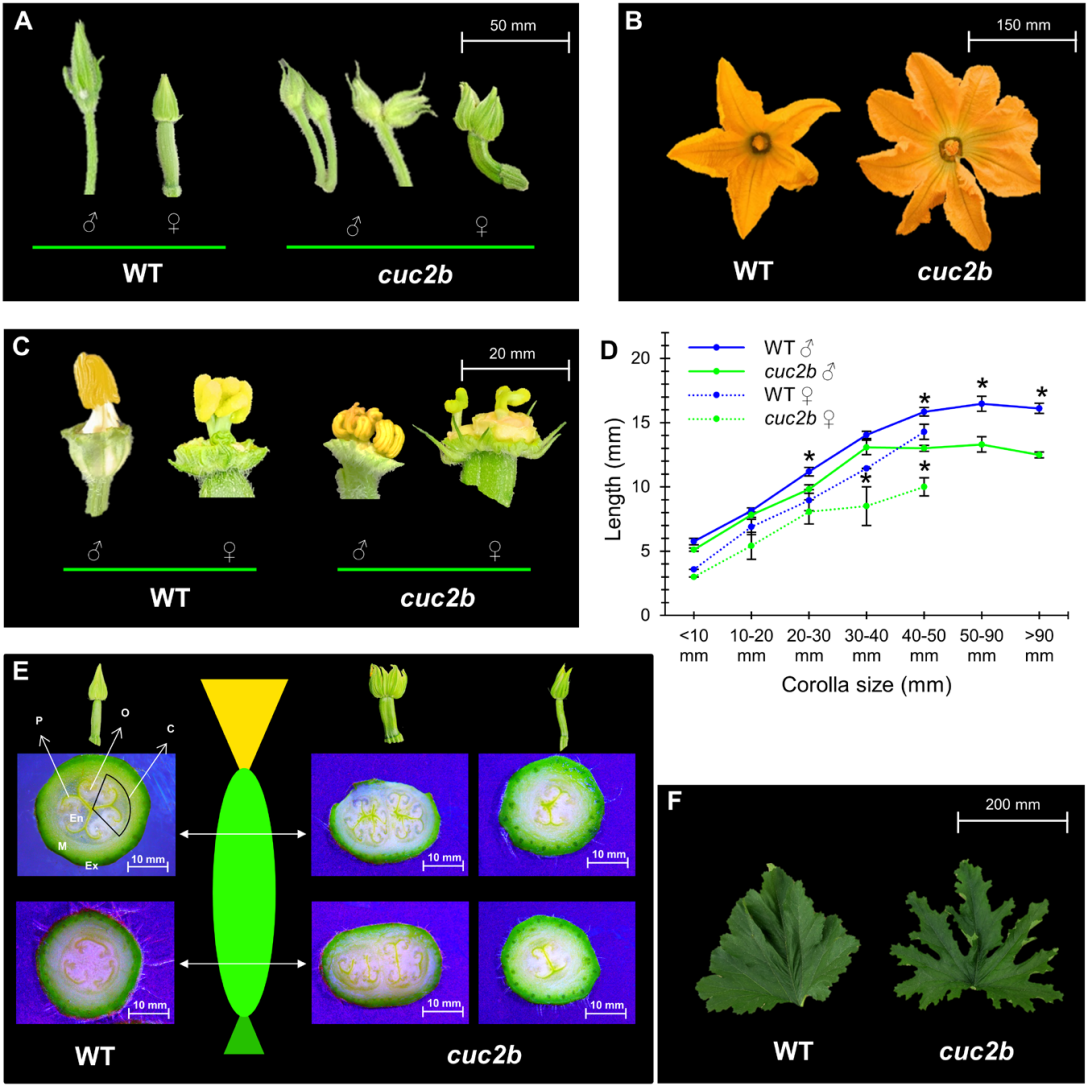
Effects of the *cuc2b* mutation on flower and leaf development. (A) Double male and female flowers in *cuc2b* nodes. (B) Increase in the number of petals in *cuc2b* flowers. (C) Alterations in the number and development of stamens and carpels. (D) Length of reproductive organs (stamen in male flowers and pistils in female flowers) relative to corolla length. Error bars represent the SE. Asterisks (*) indicate significant differences between genotypes at a specific corolla development stage (ANOVA, *P*≤0.05). (E) Transverse sections of WT and *cuc2b* ovaries in single and double flowers. Both distal sections (close to the corolla) and proximal sections (close to the pedicel) are shown. P, placenta; O, ovule; C, carpel; En, endocarp; M, mesocarp; Ex, exocarp. (F) WT and *cuc2b* leaf serration.

The *cuc2b* mutation altered the development of the floral meristem and the primordia of the lateral organs (Fig. 2). *Cucurbita pepo* plants only develop a single male or female flower in each leaf axil, and the WT of the population studied did so. However, in *cuc2b* plants, 37.4% of male nodes and 17.6% or female nodes developed two flowers (Fig. 2A; Table 2). Some of these double flowers fused along the pedicels (male flowers) or along the pedicels and ovaries (female flowers) (Fig. 2A), indicating accessory flowers derived from a very early division of the axillary floral meristem.

**Table 2.** Effect of the *cuc2b* mutation on the number of flowers per node and the number of floral organs

	Node	Double flowers (%)	No. of sepals	No. of petals	No. of stamens	No. of carpels	Bisexual flowers (%) ^*a*^
WT	♀		5.03 ± 0.003 b	5.03 ± 0.003 a		3.1 ± 0.06 b	0
	♂		5 ± 0.0 b	5 ± 0.0 a	3 ± 0.0 a		
*cuc2b* single flowers	♀		5.76 ± 0.16 c	5.81 ± 0.22 b		2.71 ± 0.26 ab	2.4
	♂		5.53 ± 0.08 c	5.60 ± 0.12 b	4.22 ± 0.11 b		
*cuc2b* double flowers	♀	17.6	3.86 ± 0.31 a	5.18 ± 0.35 ab		2.05 ± 0.30 a	2.4
	♂	37.4	4.37 ± 0.16 a	5.09 ± 0.31 a	4.10 ± 0.28 b		

^*a*^ Ovary-bearing flowers with anthers but without a style or stigma from the total of flowers analyzed of each genotype. *n*=250 male flowers and 90 female flowers. Different letters in the same column indicate significant differences between genotypes (ANOVA, *P*≤0.05).

The number and separation of floral organs differed between WT and mutant male and female flowers (Fig. 2B, C; Table 2). In single flowers, the number of sepals and petals increased from 5 in WT to 5.53 and 5.60 in the male flower of *cuc2b* and to 5.76 and 5.81 in the female flowers of *cuc2b*, respectively. The number of stamens also increased from 3 in the WT to 4.22, but the number of carpels was reduced from 3 to 2.71 carpels in the *cuc2b* mutant flowers (Table 2). In the double *cuc2b* flowers, the number of floral organs per flower varied with respect to those of the single *cuc2b* flowers (Table 2). The number of sepals and petals was reduced compared with that of the single flowers, but the number of stamens and carpels was similar in the single and double flowers of *cuc2b* (Table 2), suggesting that the division of the axillary floral meristem probably occurred after the initiation of the petal primordia, but before the initiation of stamens and carpels. Furthermore, compared with the WT, *cuc2b* flowers appeared to have a reduced stamen and pistil length, indicating that the mutation also altered the growth of sexual organs, especially during the later stages of flower development (Fig. 2D). Moreover, 2.4% of total *cuc2b* flowers were bisexual and developed both carpels and stamens, (Table 2).

The cross-sections of the single and double mutant ovaries indicated that exocarp and mesocarp develop normally, but the development of the endocarp and placenta is partially or completely abolished (Fig. 2E). In bisexual flowers, the endocarp and placenta tissue was also completely abolished. The WT pistil comprises three fused styles and stigmas, and an inferior ovary with three carpels, each developing two rows of placentas and ovules along the ovary (Fig. 2E). Although the phenotype was variable among flowers, most *cuc2b* pistils comprise fewer than three carpels at the distal end close to the flower corolla, with an average number of 2.71 and 2.05 styles, stigmas, and placentas in single and double *cuc2b* flowers, respectively. As we move towards the proximal end, the endocarp of the *cuc2b* ovary was replaced by mesocarp tissue and the number of placentas was further reduced (Fig. 2E). These flower defects caused partial female fertility, with a very reduced number of seeds compared with WT flowers (Table 3). However, the male flower of *cuc2b* was fully fertile, producing a similar number of seeds when used as a pollinator of the WT flowers (Table 3).

**Table 3.** Female and male fertility of WT, heterozygous, and homozygous *cuc2b* mutant plants

♀ Parent	♂ Parent	No. of fruits	No. of seeds	No. of seeds/fruit
wt/wt	wt/wt	30	10 000	333
wt/wt	*cuc2b/cuc2b*	7	1490	213
wt/wt	wt/*cuc2b*	1	300	300
wt/*cuc2b*	wt/wt	2	420	210
wt/*cuc2b*	wt/*cuc2b*	3	600	200
*cuc2b/cuc2b*	wt/wt	6	168	28
*cuc2b/cuc2b*	*cuc2b/cuc2b*	2	65	33

In contrast to the defects found in flower meristem specification, the apical and axillary vegetative meristems of *cuc2b* plants developed normally (Supplementary Fig. S1). The *cuc2b* leaf, however, also had defective development patterns, with the mutation enhancing the serration of the leaf margin (Fig. 2F), similar to what occurs for *miR164* mutants and *miR164*-resistant alleles of *CUC2* in Arabidopsis (Nikovics *et al.*, 2006).

### *cuc2b* disrupts the NAC-like gene *CpCUC2B* on *C. pepo* chromosome 6

**Fig. 3.**
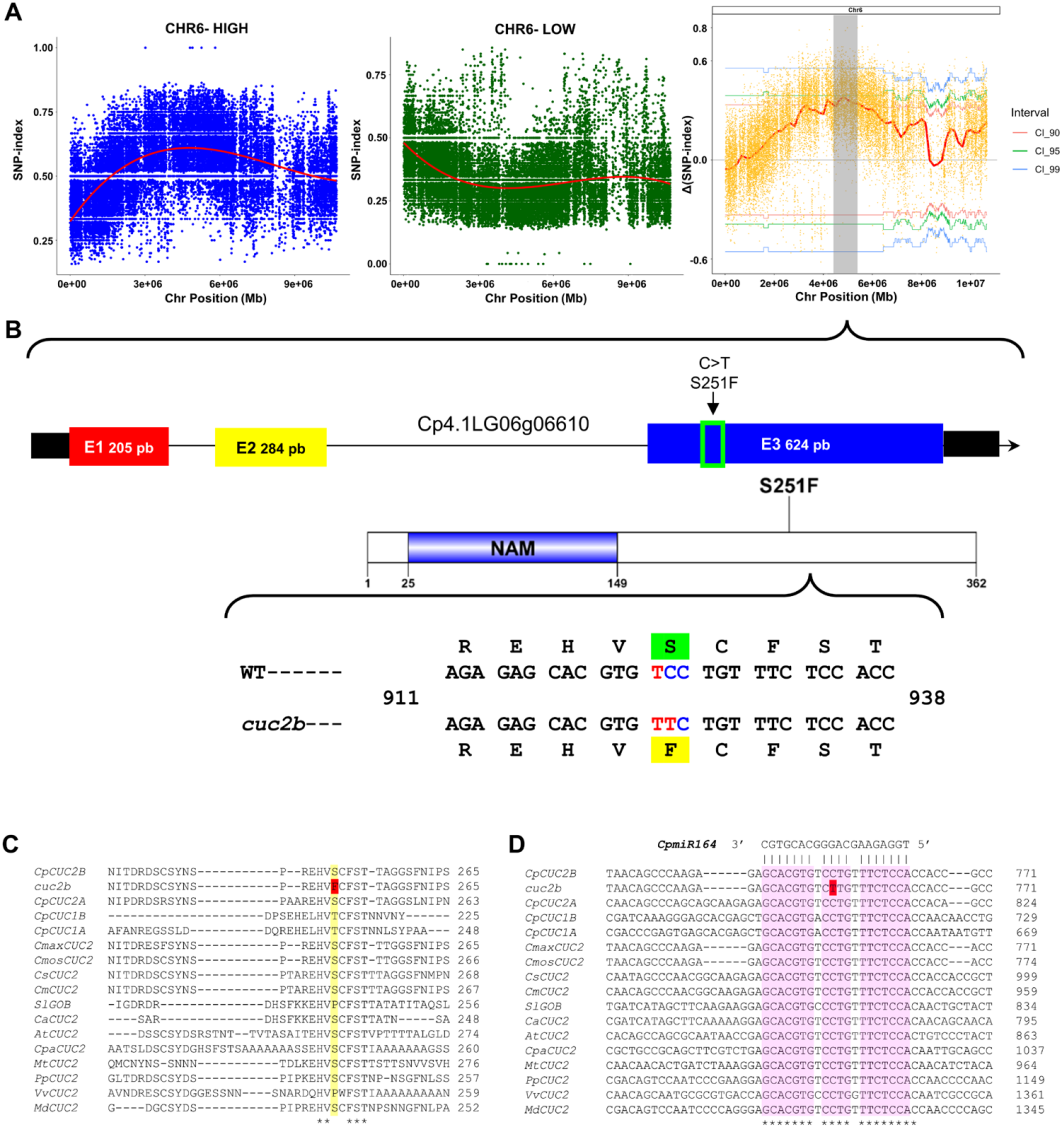
Identification of the *cuc2b* causal mutation by BSA-seq. (A) SNP index plots of WT and mutant bulks, and the ΔSNP index plot on chromosome 6. The fine mapping of this region allows the identification of an SNP in the candidate region of chromosome 6 as the causal mutation of the *cuc2b* phenotype. (B) Genomic location of the *cuc2b* mutation in exon 3 of a *NAC*-*like* gene (*Cp4.1LG06g06610*) in *Cucurbita pepo* subgenome B. The gene was called *CpCUC2B* because it is coding for a protein with a high identity to *Arabidopsis thaliana* transcription factor CUC2. (C) The *cuc2b* mutation causes a change of serine to phenylalanine in residue 251 of CpCUC2B. (D) Multiple alignment of the *miR164*-binding site in the mRNA sequence of the *CUC2* genes of different organisms (Cp, *Cucurbita pepo*; Cmax, *Cucurbita maxima*; Cmos, *Cucurbita moschata*; Cs, *Cucumis sativus*; Cm, *Cucumis melo*; Ca, *Capsicum annuum*; At, *Arabidopsis thaliana*; Cpa, *Carica papaya*; Mt, *Medicago truncatula*; Pp, *Prunus persica*; Vv, *Vitis vinifera*; Md, *Malus domestica*). The *cuc2b* mutation hits a very conserved nucleotide of the *miR164*-binding site of *CpCUC2B*.

To elucidate the causative mutation of the *cuc2b* phenotype, the mutant was crossed twice with the zucchini cultivar MU-CU-16 (background genotype of the EMS population), and the obtained BC2 generation was selfed to generate a BC2S1 segregating population. The *cuc2b* mutant was also crossed with the scallop cultivar UPV-196 and then selfed to generate an F_2_ segregating population. The BC2S1 population on the MU-CU-16 background was used for the phenotyping of mutants, as the two backcrosses reduced the number of EMS mutations other than *cuc2b*. On the other hand, the F_2_ population was used for BSA-seq. A total of 15 WT and 15 *cuc2b* F_2_ plants were separated in two DNA bulks (WT and *cuc2b*) and used for WGS. More than 80 million reads were obtained that covered >97% of the reference genome with an average depth of 40 and 37.21 in the WT- and *cuc2b*-bulk, respectively (Table 4). The 1.51 and 1.49 million SNPs in the WT- and mutant-bulk were used to run the QTLseqr package, calculating and plotting the SNP index and ΔSNP index against the position of the genome. This allowed the identification of a single region on chromosome 6 where the average SNP index was higher in the WT-bulk than in the mutant-bulk (Fig. 3A). The ΔSNP index values in this region of chromosome 6 (position 2 982 470 to 5 702 905) differed significantly from 0, indicating this region harbors a major QTL (QTL6) that is probably responsible for the *cuc2b* phenotype (Fig. 3A).

**Table 4.** Summary of sequencing data and filtering for SNPs in WT and *cuc2b* DNA bulks

Whole-genome sequencing data					WT	*cuc2b*
No. of reads					90 123 030	83 373 064
Mapped reads (%)					97.87	97.42
Average depth					40.00	37.21
Coverage at least 4× (%)					95.65	94.99
**SNPs**						
No. of SNPs					1 513 525	
EMS SNPs (G>A/C>T)					141 960	
AF (WT-bulk) <0.35; AF (*cuc2b-*bulk) >0.65					2888	
Exonic SNPs					263	
Non-common SNPs					97	
EMS SNPs (GQ >90; DP >10)					39	
Non-synonymous SNPs					16	
Non-synonymous SNPs on QTL6					2	
**Candidate SNPs**						

Chr	Position	Ref	Alt	Gene ID	Effect	Annotation
6	4168895	C	T	Cp4.1LG06g06610	S230F	NAC domain protein
6	4671155	G	A	Cp4.1LG06g08960	V884M	Autophagy-related protein 18f-like

AF, allelic frequency; GQ, genotype quality; DP, read depth.

Although the two bulks were made up of the most extreme phenotypes, given that the *cuc2b* mutant was semi-dominant, its causal mutation should have a minimum SNP index of 0.66 in the mutant-bulk and a maximum SNP index of 0.33 in the WT-bulk. Therefore, the >141 000 canonical EMS mutations (C>T or G>A) identified were filtered by their allele frequency (AF>0.65 in the *cuc2b*-bulk and AF<0.35 in the WT-bulk), which resulted in 2888 mutations. Only 97 of the filtered mutations were exonic and absent from other lines in the mutant collection, 16 were non-synonymous, and only two of them were located in QTL6 (SNP1: Cp4.1LG06:4168895 and SNP2: Cp4.1LG06:4671155). SNP1 was a C>T transition in the gene *Cp4.1LG06g06610*, which encodes a transcription factor containing the NAC domain (Table 3), while SNP2 was a C>T transition in the gene *Cp4.1LG06g08960*, which encodes the 18f-like protein related to autophagy (Table 4).

To confirm the causal mutation of the *cuc2b* phenotype, a total of 177 plants from a BC1S1 population were genotyped for both SNP1 and SNP2 in QTL6. Only SNP1 co-segregated perfectly with the *cuc2b* locus, but 13% recombination was found between *cuc2b* and SNP2 (Supplementary Dataset S1). Furthermore, we genotyped two additional segregating populations, 179 plants from a BC1S1 generation and 280 plants from a BC2S1 generation, finding a perfect co-segregation between SNP1 and the *cuc2b* phenotype (Supplementary Dataset S1). Based on the co-segregation between SNP1 and the mutant phenotype in 636 plants, we concluded that the *CUC2B* locus is probably encoding the NAC transcription factor gene *CpCUC2B*.

The mutation *cuc2b* affected the third exon of the gene, producing a C>T transition at the 753 nucleotide position of the *CpCUC2B* transcript, and a serine to phenylalanine substitution at position 251 of the protein (S251F) (Fig. 3B). When homologous protein sequences from different plants were aligned, it was found that S251 was not a very conserved residue between plants, and bioinformatic analysis with the SNAP2 tool predicted a reduced effect of S251F substitution on protein function (Fig. 3C; Supplementary Figs S2, S3). However, the 21 nucleotide sequence around the *CpCUC2B* mutation was highly conserved in plants. This conserved region of mRNA corresponds to the *miR164*-binding site (Fig. 3D), a negative post-transcriptional regulator of these NAC-like transcription factors in different plant species (Mallory *et al.*, 2004; Adam *et al.*, 2011) (Supplementary Fig. S4). The mutation affects nucleotide 10 of the binding site in mRNA, which is part of the *miR164* slicing site (Voinnet, 2009).

### Phylogeny and molecular structure of *CpCUC2B*

**Fig. 4.**
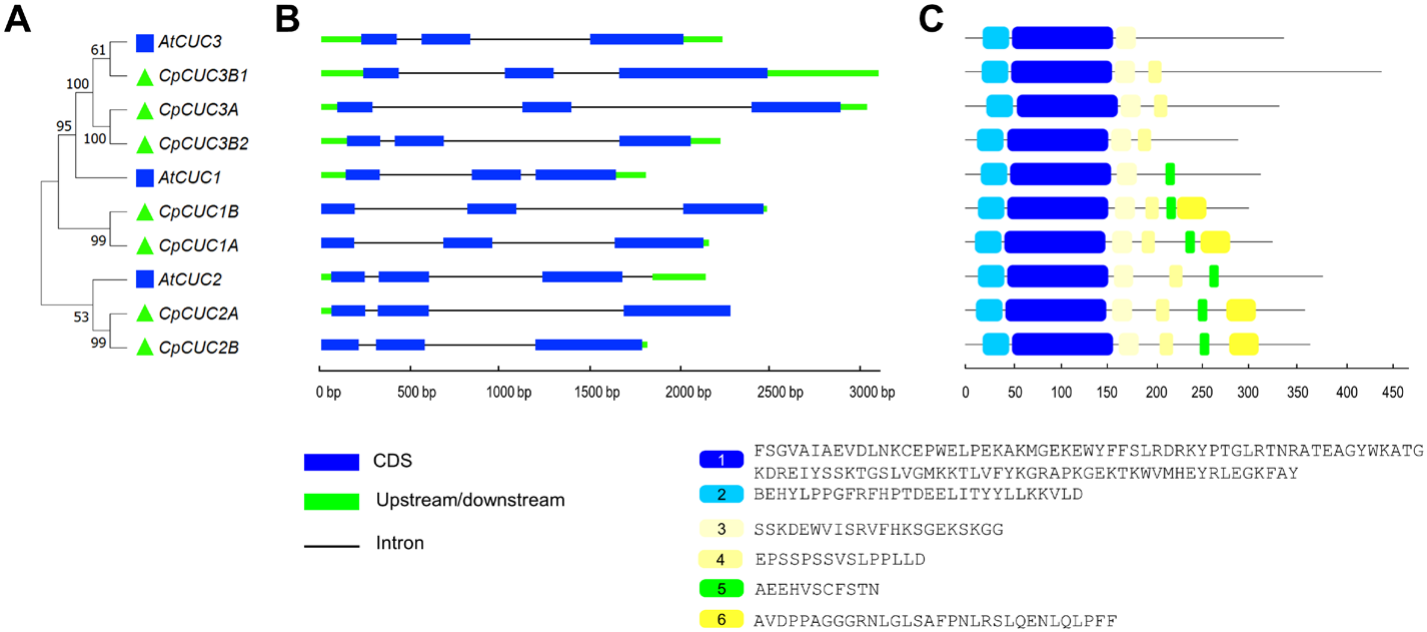
Phylogenetic relationships, gene structure, and architecture of conserved protein motifs in *CUC* genes from *Cucurbita pepo* and *Arabidopsis thaliana*. (A). Phylogenetic tree constructed based on the CUC full-length protein sequences. (B) Exon–intron structure of *CUC* genes. (C) Schematic representation of the conserved motifs in CUC proteins. Motifs 1 and 2 comprise the NAC domain of this family of transcription factors. Motifs 4, 5, and 6 correspond to motifs I, II, and III in Adams *et al*. (2011).

An NCBI BLASTn and BLASTp search with nucleotide and amino acid sequences of *Cp4.1LG06g06610* demonstrated a high homology with Arabidopsis *CUP-SHAPED COTYLEDON* clade genes *CUC1*, *CUC2*, and *CUC3*, three transcription factor genes of the NAC family. We searched for other *CUC* genes in the *C. pepo* genome. Since the genome of *C. pepo* is duplicated (Sun *et al.*, 2017; Montero-Pau *et al.*, 2018), we found seven *CUC* homologs. The clustal analysis performed with the three Arabidopsis and the seven squash CUC proteins indicated that *Cp4.1LG06g06610* probably corresponded to Arabidopsis *CUC2* (Fig. 4A) and, since it is located in the B subgenome, the gene was called *CpCUC2B*. Its paralog in subgenome A, *CpCUC2A* (*Cp4.1LG02g01950*), was found in a syntenic block between chromosomes 6 and 2 (Cucurbit Genomic Database, http://cucurbitgenomics.org/). The two paralogs shared >80% homology. The other five squash *CUC* genes were clustered with Arabidopsis *CUC1* or *CUC3* (Fig. 4A), suggesting that all these gene functions are conserved between Arabidopsis and squash.

The *CpCUC2B* and *CpCUC2A* paralogs, but also the other *CUC-like* squash genes, were found to have the same molecular structure as those of Arabidopsis and other species, with three exons and two introns (Fig. 4B), as well as the same domain architecture as CUC-like transcription factors (Fig. 4C). The *miR164*-binding site was only found in the *CpCUC1* and *CpCUC2B* transcripts, indicating a common post-transcriptional regulation of the *CUC1* and *CUC2* genes in Arabidopsis and squash.

The analysis of conserved motifs using the MEME tool (https://meme-suite.org/meme/tools/meme) allowed the identification of six different motifs, the same domain architecture as CUC-like transcription factors (Fig. 4C). Motif 1 and motif 2 correspond to the conserved non-apical-meristem (NAM) domain, characteristic of NAC genes in this category. Motif 4 corresponds to motif I in Adam *et al.* (2011) and comprised the L motif in Larsson *et al.* (2012). Motifs 5 and 6 were specific to CpCUC1 and CpCUC2 and were absent in CpCUC3. Motif 5, defined as motif II in Adam *et al.* (2011) or motif V in Larsson *et al.* (2012), corresponds to the *miR164*-binding site in the *CUC* mRNA sequence, while the function of motif 6, defined as motif III in Adam *et al.* (2011), has not been determined yet (Maugarny *et al.*, 2016).

### Tissular regulation of the *C. pepo miR164* and *CUC* genes

Since *CUC1* and *CUC2* expression is known to be regulated by *miR164* in Arabidopsis and other species, a search was conducted to identify potential loci transcribing this specific miRNA. Six *miR164* loci were identified in the *C. pepo* genome, located on chromosomes 3, 8, 4, 10, 18, and 19, respectively (Supplementary Fig. S5). The *MIR164C1* transcript (LOC111803168 on chromosome 10) and the *MIR164C2* transcript (LOC111782191 on chromosome 19) were previously annotated as non-coding RNAs (ncRNAs) consisting of 410 and 1088 nucleotides, respectively, in the NCBI database (Supplementary Fig. S5). The loci *MIR164A1*, *MIR164A2*, *MIR164B1*, and *MIR164B2* were also identified in our *C. pepo* RNA-seq projects, which included transcripts of 421, 399, 410, and 210 bp, respectively (Supplementary Fig. S5).

**Fig. 5.**
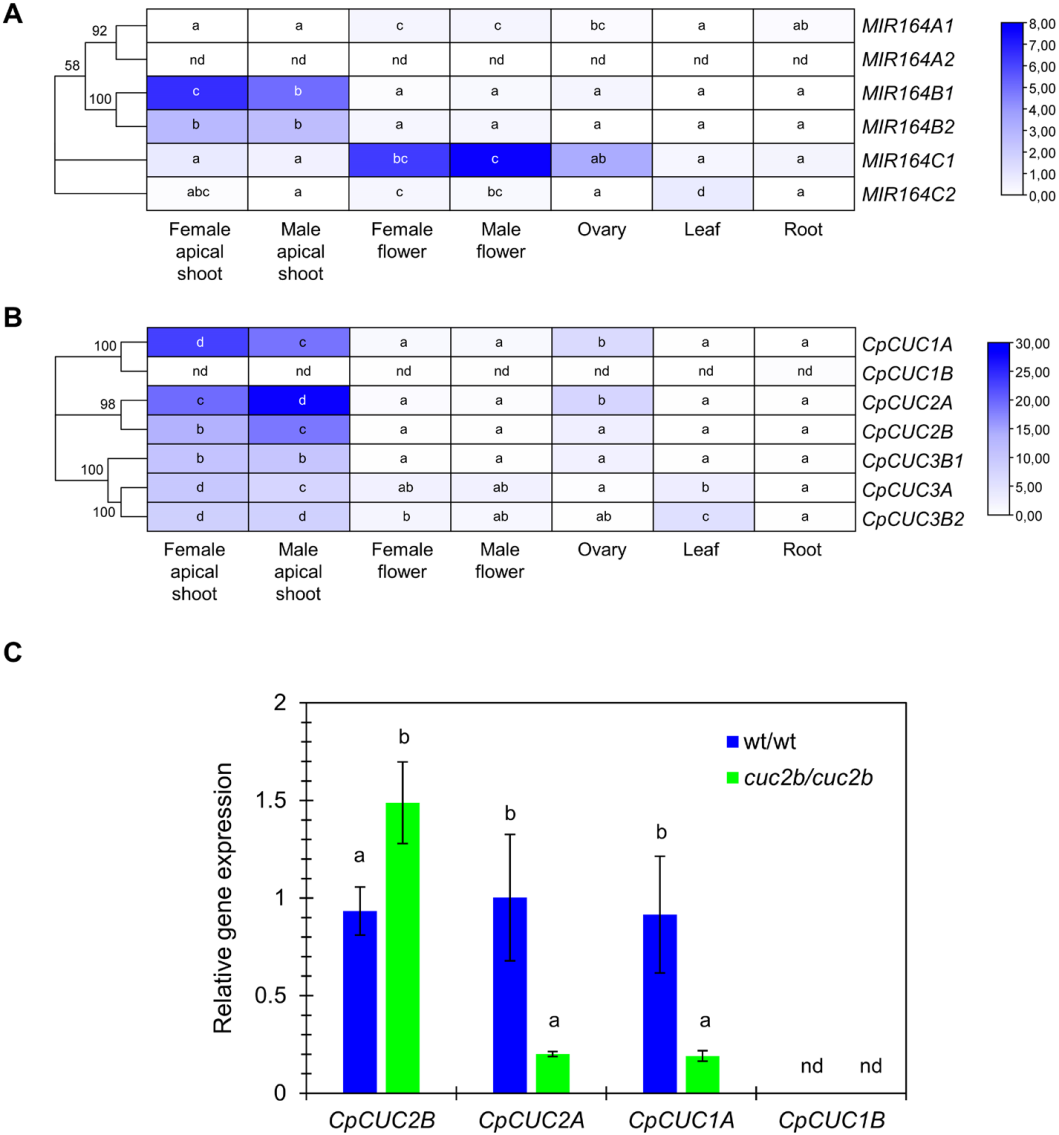
Expression patterns of (A) *MIR164* and (B) *CpCUC* genes in different organs of WT plants: apical shoot at the three (male apical shoot before female flowering) and 12 nodes stage (female apical shoot after female flowering), male and female flowers with a corolla length of 30 mm, ovaries from flowers at the same developmental stage, and leaves of 10 mm and roots from plantlets 17–21 d after germination. FPKM values were used to generate the heatmap with hierarchical clustering analysis. The scale represents the relative signal intensity of the FPKM values. (C) qRT-PCR of *CpCUC* genes in the apical shoots of WT and *cuc2b* plants after female flowering. PCR primers for *CpCUC* genes were designed in the flanking regions of the predicted *miR164*-binding sites. Different letters indicate significant differences between samples for each gene (ANOVA, *P*≤0.05); nd, no detectable expression.

RNA-seq data from different plant organs revealed that the highest expression of *MIR164* genes was in the apical shoots and in female and male flowers, including the ovary (Fig. 5A). *MIR164C1* was detected in all plant organs, with higher expression levels in male and female flowers (flowers with a 30 mm length corolla). *MIR164C2* exhibited higher expression in leaves and in female and male flowers, but appeared to have reduced expression in other organs. *MIR164B1* showed predominant expression in the apical shoots, but reduced expression in other organs, and the same was true for *MIR164B2*, but with a lower expression level. *MIR164B1* showed higher expression in the apical shoots of plants after female flowering (female apical shoots) than in the apical shoots of plants before female flowering (male apical shoots), suggesting the involvement of this *miR164b1* in the regulation of the female flowering transition (Fig. 5A). *MIR164A2* did not show detectable expression in the tissues analyzed, while *MIR164A1* showed reduced expression compared with the other *MIR164* loci (Fig. 5A).

The same RNA-seq data also revealed that, in general, the transcription of *CUC* genes was negatively correlated with *MIR164* expression (Fig. 5B). The *CUC* genes were expressed mainly in the apical shoots of plants producing male and female flowers (Fig. 5B). They were also found to be expressed in the ovary and the leaf, but showed reduced expression in female and male flowers (Fig. 5B). *CpCUC2A* and *CpCUC2B* showed a high expression in the apical shoots producing both lateral male flowers (male apical shoots) or female flowers (female apical shoots), with their expression negatively correlated with the abundance of *MIR164B1* transcripts, namely higher in the apical shoots of plants before female flowering, and therefore producing only male flowers (Fig. 5B). Female flowering was then related to an up-regulation of *MIR164B1* and a down-regulation of *CpCUC2A* and *CpCUC2B*. The ovaries also exhibited high expression of the genes *CpCUC1A*, *CpCUC2A*, and *CpCUC2B*, although the values were lower than in the apical shoots. In male and female flowers, leaves, and roots, *CUC* genes showed very low expression levels (Fig. 5B). The *CpCUC1B* transcript was not detectable in the different tissues analyzed (Fig. 5B).

Given the remarkable expression of *CpCUC* genes in the apical shoot, qRT-PCR was used to compare the relative expression of *CpCUC2B*, but also *CpCUC2A*, *CpCUC1A*, and *CpCUC1B*, in the apical shoots of WT and *cuc2b* plants after the female flowering transition (Fig. 5C). Given that these genes could be regulated by *miR164*, primers for gene expression were designed in the flanking regions of the predicted *miR164*-binding site, so detecting only the non-degraded mRNA. It was confirmed that *CpCUC1B* is not expressed in the apical shoots. The *CpCUC2B* gene was found to be overexpressed in the apical shoots of *cuc2b* plants relative to the WT (Fig. 5C). These results indicate that *cuc2b* is likely to be a *miR164*-resistant allelic variant that increases *CpCUC2B* transcription, which explains the mutant gain-of-function phenotype. To validate the predicted binding and cutting site of miR164, relative gene expression was also assessed using primers designed in the 3' region of the transcript, outside the *miR164*-binding site (Supplementary Fig. S6). With the use of the latter primers, the level of *CpCUC2B* transcripts was the same in WT and *cuc2b* plants (Supplementary Fig. S6). Therefore, the differences in expression between WT and *cuc2b* with the primers flanking the *miR164*-binding site would indicate a reduced integrity of the WT transcript in that region, confirming the *miR164* binding and cutting site. On the other hand, the *cuc2b* mutation diminished the expression of *CpCUC2A* and *CpCUC1A* in the apical shoot of plants, suggesting a possible increased competitive action of *miR164* on these two genes (Fig. 5B).

### Regulatory network of *CpCUC* genes

To elucidate the different elements that may regulate *CUC* genes in response to various stresses or hormones, *cis*-regulatory elements (CREs) in the promoter region of Arabidopsis and squash *CUC* genes were identified by searching in a 1000 bp region upstream of the ATG start codon (Supplementary Fig. S7A). The presence of regulatory elements that respond to abiotic stresses was notably observed, highlighting the importance of *CUC* genes in environmental stress responses. Moreover, *CUC* genes appear to respond to MYB-, MYC-, and WRKY-like transcription factors, all of them involved in stress response. Elements responsive to jasmonic acid (JA) and gibberellins (GAs) were found in the *CpCUC2B* promoter, while its paralog *CpCUC2A* was found to be responsive to ethylene, JA, and abscisic acid (ABA) (Supplementary Fig. S7A).

The transcriptomic data of *C. pepo* obtained by our group in different RNA sequencing projects was used to predict the potential regulatory network of *CpCUC1* and *CpCUC2*, the *miR164*-regulated CUC genes. We found that there are 99 genes regulated by *CpCUC2A*, 90 by *CpCUC2B*, and 48 by *CpCUC1A* (Supplementary Dataset S2). *CpCUC2B*, *CpCUC2A*, and *CpCUC1A* appear to regulate several meristem-determining genes such as *CpSOL2/CRN*, *CpCLAVATA2*, and *CpSQN*, whose mutants in Arabidopsis exhibit alterations in the number of carpels (Müller *et al.*, 2008). They also regulate certain homeodomain protein genes (*CpAH22*, *CpPDF2*, and *CpARID1*) that affect embryonic and gamete development (Tan and Irish, 2006; Ogawa *et al.*, 2015; Li *et al.*, 2017). The expression of *CUC* genes was also related to hormone transduction pathways, particularly those involved in ABA or auxin signaling. Regarding organ boundary maintenance, the presence of two MYB-like transcription factor genes, *CpMYB105* and *CpMYB117*, with significant effects on organ patterning in Arabidopsis is important (Lee *et al.*, 2009; Gomez *et al.*, 2011). *CpCUC2B* appears to also regulate *CpWIP1B*, encoding a key transcription factor orchestrating the development of male flowers in cucurbits (Supplementary Fig. S7B).

### Crosstalk between *CpCUC2B* and ethylene in flower development and sex determination

Given that ethylene is the main regulator of sex determination in cucurbits, we studied the crosstalk between *CpCUC2B* and ethylene in the control of sex determination. Three different approaches were used for this purpose: (i) determining the phenotype of double mutants between *cuc2b* and *aco1a* and *etr2b*, two mutations affecting ethylene biosynthesis and signaling, respectively; (ii) comparing the expression of ethylene genes in WT and *cuc2b*; and (iii) comparing the expression of *CUC* and *MIR164* genes in the WT and the ethylene-deficient and insensitive mutants *aco1a*, *etr1a-1*, *etr1b*, and *etr2b*.

The flower phenotypes of single and double mutants *cuc2b*/*aco1a* and *cuc2b/etr2b* are shown in Table 5 and Supplementary Fig. S8. Both *aco1a* and *etr2b* exhibited a partial andromonoecious phenotype (García *et al.*, 2020b; Cebrián *et al.*, 2022), with 25.9% and 2.5% of total flowers converted into bisexual or hermaphrodite flowers in *aco1a* and *etr2b*, respectively (Table 5). Therefore, both *CpACO1A* and *CpETR2B* are involved in the arrest of stamens during the development of female flowers. Like *cuc2b*, the single *etr2b* also showed a decrease in the number of pistillate flowers per plant (7.6%), indicating that *etr2b* directly converted a large number of female flowers into male flowers (Table 5). The ethylene response mediated by *CpETR2B* is therefore involved not only in the arrest of stamen but also in the promotion of carpel development in female flowers. This is contrary to the function of *CpCUC2B*, which, as demonstrated by the maleness phenotype of the gain-of-function *cuc2b*, acts as a repressor of carpel development for the specification of male flowers. However, single *aco1a* and *etr2b* mutants did not show the ectopic floral meristem or ectopic floral organs of *cuc2b*, producing only one flower per node and five sepals and petals, and three stamens or carpels per male of female flower, respectively (Table 5).

**Table 5.** Phenotypic evaluation of single and double mutants between *cuc2b* and the ethylene-deficient and ethylene-insensitive mutants *aco1a* and *etr2b*

	Node	Double flowers (%)	Flowers with changes in the number of floral organs with respect to the WT (%)				Bisexual flowers (%)^*a*^	Pistillate flowers (%)
			Sepals	Petals	Stamens	Carpels		
wt/wt	♀	0	0	0		0	0	23.7 ± 1.2
	♂	0	0	0	0			
*cuc2b*	♀	17.6	76	63		72	2.4	7.6 ± 1.1
	♂	37.4	69	60	81			
*aco1a*	♀	0	0	0		0	25.9	35.01 ± 3.4
	♂	0	0	0	0			
*etr2b*	♀	0	0	0		0	2.5	7.84 ± 1.35
	♂	0	0	0	0			
*cuc2b/aco1a*	♀	0	67	33		12	2.6	6.35 ± 2.15
	♀	17	32	65	39	–		
*cuc2b/etr2b*	♀	–	–	–			–	0.0 ± 0.0
	♀	26	65	40	56			

^*a*^ Ovary-bearing flowers with anthers from the total of flowers analyzed of each genotype.

The homozygous double mutant *cuc2b/aco1a* showed a similar reduction in the percentage of pistillate flowers per plant (6.35%) to *cuc2b* (7.6%) (Table 5), and very different from the one shown by *aco1a* (35.01%), indicating that *cuc2b* is epistatic over *aco1a* in the control of sex determination. A total of 2.6% of the total flowers in the double mutant were bisexual (Supplementary Fig. S8), similar to the *cuc2b* single mutant, indicating a slight lack of stamen arrest in those floral meristems determined as female flowers, but the reduced number of female flowers in the double mutant *cuc2b/aco1a* and the environmental dependence of the stamen development phenotype made it difficult to establish a solid correlation between the effect of both mutations in the phenotype. The specific phenotypic defects of *cuc2b* were more pronounced in male flowers than in female double mutant flowers. Therefore, the percentage of affected flowers was lower in *cuc2b/aco1a* than in *cuc2b* (Table 5). Thus, double flowers occurred in male but not in female nodes of the double mutant, and changes in the number of floral organs affected male flowers more than female flowers (Table 5).

On the other hand, the homozygous double mutant *cuc2b/etr2b* was completely androecious and produced no pistillate flowers at all. The higher severity of the *cuc2b/etr2b* phenotype compared with that of the single mutants, with complete suppression of carpel development and complete conversion of female into male flowers, indicates a synergistic effect of *cuc2b* and *etr2b* on sex determination. Male flowers of *cuc2b/etr2b* showed the specific defects of the single mutant *cuc2b*, with double flowers and a higher number of sepals, petals, and stamens than in the WT (Table 5).

**Fig. 6.**
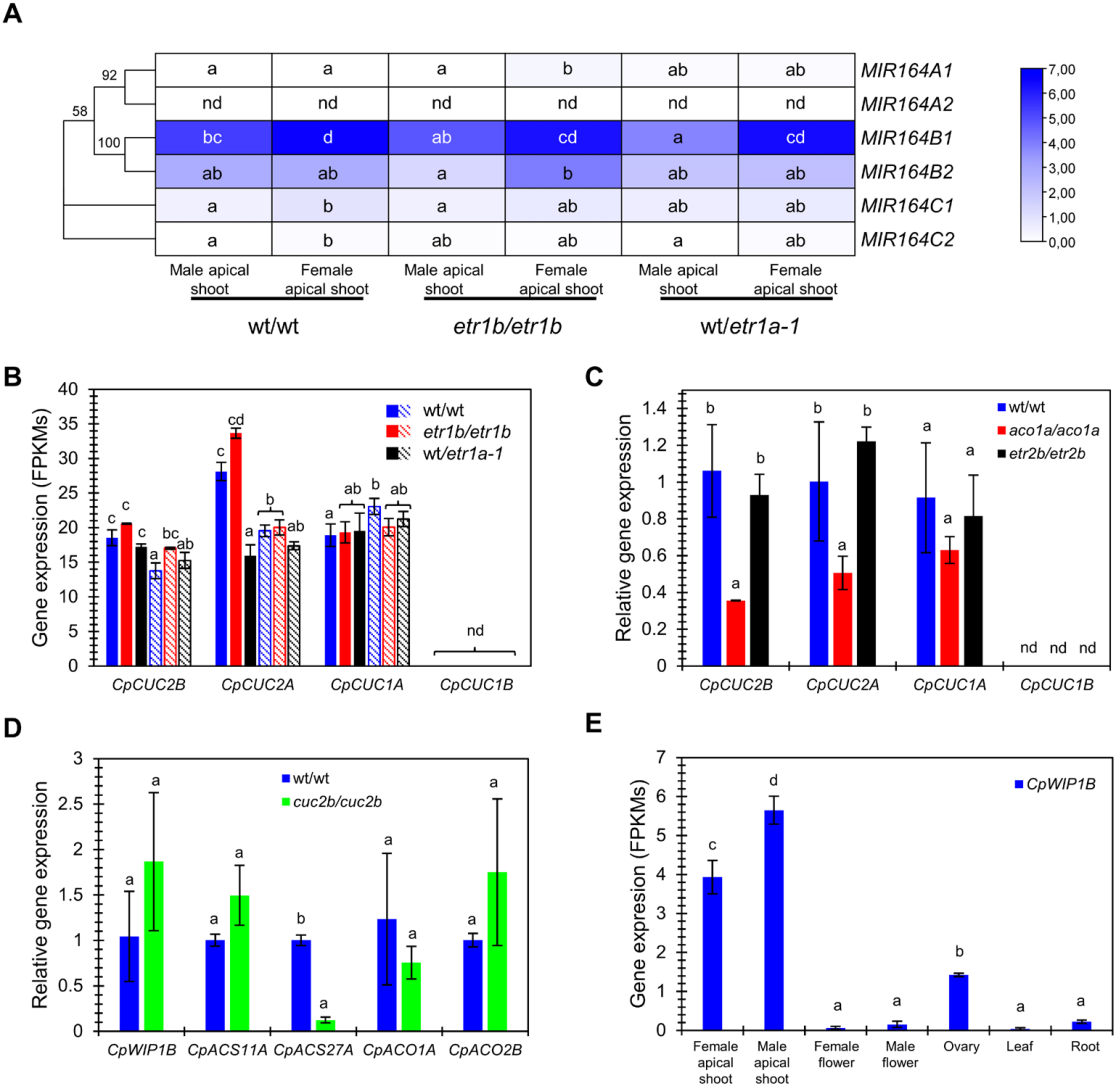
Crosstalk between ethylene and *CpCUC* genes in the regulation of sex determination in *C. pepo*. Gene expression of *miR164* and *CpCUC* genes in ethylene-deficient and insensitive mutants, and of ethylene and other sex-regulating genes in the *cuc2b* mutant is shown. (A) Expression patterns of *MIR164* loci and (B) *CpCUC* genes regulated by *miR164* in the apical shoots of the ethylene-insensitive androecious mutants *etr1b* and *etr1a-1* plants at the three nodes (male apical shoot, solid color) and 12 nodes stage (female apical shoot, striped pattern color). FPKM values were used to generate the heatmaps with hierarchical clustering analysis. The scale represents the relative signal intensity of the FPKM values. (C) Relative gene expression obtained by qRT-PCR of *CpCUC* genes regulated by *miR164* in the apical shoots of WT and ethylene-deficient (*aco1a*) and ethylene-insensitive (*etr2b*) mutants. (D) Relative gene expression obtained by qRT-PCR of ethylene biosynthesis genes and the transcription factor gene *CpWIP1B* in the apical shoots of WT and *cuc2b* plants. (E) Expression patterns of *CpWIP1B* in different organs of WT plants: apical shoot at the three (male apical shoot, before female flowering) and 12 nodes stage (female apical shoot, after female flowering), male and female flowers with a corolla length of 30 mm, ovaries from flowers at the same developmental stage, and leaves of 10 mm and roots from plantlets of 17–21 d after germination. FPKM values were used to generate the graph. Different letters indicate significant differences between samples in each gene (ANOVA, *P*≤0.05). nd, no detectable expression.

In Arabidopsis, ethylene is known to regulate the expression of the *miR164* and *CUC* genes (Li *et al.*, 2013). To investigate whether ethylene can also modulate the expression of the *CUC*/*miR164* module in the apical shoot of squash, its expression was comparatively studied in WT and ethylene-deficient (*aco1a*) and ethylene-insensitive (*etr1a-1*, *etr1b*, and *etr2b*) mutants. Both *etr1b* and *etr1a-1* confer an androecious phenotype, and plants only produced male flowers (García *et al.*, 2020a). RNA-seq data from apical shoots of plants in the male and female stages of development confirmed that the *MIR164B1* or *MIR164B2* loci (highly expressed in the apical shoots of both WT and mutant plants) were up-regulated in the apical shoots of plants producing female flowers, but ethylene-insensitive mutations *etr1b* and *etr1a-1* did not produce a significant change in their expression in comparison with the WT at the same stage of development (Fig. 6A).

The *CpCUC1A* and *CpCUC1B* genes were not regulated by female flowering, nor by ethylene-insensitive mutations *etr1a-1* and *etr1b* (Fig. 6B). However, *CpCUC2A* and *CpCUC2B* were down-regulated in the apical shoots of plants producing female flowers relative to those in earlier stages, when the plant is only producing male flowers (Fig. 2B). This regulation could be mediated by *miR164*, as the *MIR164B1* gene is induced in the apical shoot upon female flowering. Only *etr1a-1* was found to down-regulate *CpCUC2A* in the male phase of development, validating the specific ethylene regulation site predicted by ARACNE in *CpCUC2A* (Fig. 6B). However, this regulation does not appear to have anything to do with sex expression, as at this stage both WT and *etr1a-1* were producing male flowers. No transcriptional changes were found for *CpCUC* genes in the apical shoots of ethylene-insensitive mutants at the female phase of development (Fig. 6B), when the WT were producing female flowers, but the ethylene mutants were still producing male flowers.

The qPCR expression analysis with primers at the *miR164*-binding site confirmed that the *CpCUC* transcripts accumulated similarly in the apical shoots of both the WT and the ethylene-insensitive mutant *etr2b* (Fig. 6C). However, the ethylene mutation *aco1a* reduced the accumulation of *CpCUC2A* and *CpCUC2B* transcripts in the apical shoots of the plant in the female phase of development (Fig. 6C). The mutations *aco1a* and *etr2b* produce an andromonoecious sex phenotype, consistent with a reduction in ethylene production or ethylene sensitivity in the lateral floral meristems of the apical shoot, and the suppression of stamen arrest in female flowers (García *et al.*, 2020b; Cebrián *et al.*, 2022). Therefore, the down-regulation of *CpCUC2A* and *CpCUC2B* in *aco1a* must occur at later stages of flower development, when the floral meristem is already determined as a female flower.

The transcription of ethylene biosynthesis sex-determining genes was finally compared in the apical shoots of WT and *cuc2b* plants at the female stage of development (Fig. 6D). Only the ethylene biosynthesis gene *CpACS27A* showed a significant down-regulation in *cuc2b* compared with the WT. CpACS27A is the partner enzyme of CpACO1A in the production of late ethylene required for stamen arrest in female flowers, which explains the development of bisexual flowers in *cuc2b*. None of the ethylene biosynthesis genes involved the promotion of carpels at earlier stages of female flower development (*CpACS11A* and *CpACO2B*) changed its expression in *cuc2b* (Fig. 6D), indicating that *CpCUC2B* suppresses carpel development on a pathway other than that of ethylene. Therefore, the phenotypes of the double mutants *cuc2b/etr2b* and *cuc2b/aco1a* and the gene expression data indicate that *CpCUC2B* and ethylene act on two independent pathways controlling the development of carpels, but interact within the same pathway for the regulation of stamen development.

The function to *CpCUC2B* is similar to that of the transcription factor WIP1, which aborts the development of carpels for the specification of male flowers. However, *CpCUC2B* is not negatively regulated by ethylene that promotes carpel development (Fig. 6B, C), as occurs for *WIP1* of melon and cucumber. The squash *CpWIP1B* is not regulated by *CpCUC2B* (Fig. 6D), indicating that *CpCUC2B* performs its function independently of Cp*WIP1B*. RNA-seq data showed that *CpWIP1B* is not specifically expressed in male flowers, but is also expressed in female flowers and in other plant organs (Fig. 6E). However, *CpWIP1B* was found to have a higher expression in the apical shoots of plants producing male flowers than in the apical shoots of plants producing female flowers (Fig. 6E), which may indicate a conserved role in the determination of squash male flowers.

## Discussion

### *cuc2b* is an *miR164*-resistant gain-of-function mutation in *CpCUC2B*

The squash mutant *cuc2b* has been isolated from a direct screening of an EMS population of squash (García *et al.*, 2018). Both QTL-seq analysis and fine mapping have shown that *cuc2b* is a canonical EMS transition C>T that produces an S251F change in the transcription factor CpCUC2B. So, the mutation was also called *cuc2b*. The gene *CpCUC2B* and its paralog *CpCUC2A* share high homology with another five genes in the duplicated genome of squash (*CpCUC1A*, *CpCUC1B*, *CpCUC3A*, *CpCUC3B1*, and *CpCUC3B2*), all of them coding for NAC-domain transcription factors, represented by the petunia *NO APICAL MERISTEM* (*NAM*), Antirrhinum *CUP*, and the three Arabidopsis *CUC1*, *CUC2*, and *CUC3* genes (Souer *et al.*, 1996; Aida *et al.*, 1997; Weir *et al.*, 2004). Based on the sequence of the NAC domain, but also on their *miR164* post-transcriptional regulation, different plant NACs are divided into two clades (Maugarny *et al.*, 2016). The first clade, represented by *NAM*, *CUC1*, and *CUC2*, along with squash *CpCUC1A/B* and *CpCUC2A/B*, displays a binding site for *miR164*. The second clade is represented by *CUC3* and, like the three squash *CpCUC3* genes, is not regulated by *miR164*. The tissular expression patterns of the six squash *MIR164* genes and the seven *CpCUC* genes found in the genome of *C. pepo* confirmed that *CUC*/*miR164* is a conserved developmental regulation module in both angiosperms and gymnosperms (Adam *et al.*, 2011; Vialette-Guiraud *et al.*, 2011). The negative regulation of squash *CpCUC1* and *CpCUC2* by *miR164* was evident in the apical shoots of plants during the female flowering transition, where *MIR164* (especially locus *MIR164B1*) was found to be up-regulated and *CpCUC2A* and *CpCUC2B* down-regulated (Fig. 5). The expression of the *CpCUC1* and *CpCUC2* genes was also negatively correlated with the expression of the *MIR164* loci in different plant organs (Fig. 5), demonstrating that these squash genes are not only those showing the highest sequence identity with Arabidopsis *CUC1* and *CUC2*, but are also regulated by *miR164*.

Different pieces of evidence indicate that squash *cuc2b* is an *miR164-*resistant and gain-of-function mutation in *CpCUC2B*. First, the change produced by the *cuc2b* mutation affects a non-conserved residue of the protein sequence but, at a nucleotide level, the transition C>T affects position 10 of the binding site of *miR164*, which is a known cutting site position for the post-transcriptional regulation of target mRNA (Voinnet, 2009). Second, the effects of this mutation, which leads to accessory floral meristems and floral organs, suppressed carpel development, and increased leaf serration, resembled those of mutants at *MIR164* loci or *miR164*-binding sites of *CUC1* and *CUC2* in Arabidopsis mutants (Laufs *et al.*, 2004; Mallory *et al.*, 2004; Baker *et al.*, 2005; Larue *et al.*, 2009) as well as the tomato *Gob4-d* (Berger *et al.*, 2009). Third, in the apical shoot, where axillary floral meristems are determined as male or female flowers, and floral organs and leaves are defining their boundaries, the complete transcripts of *CpCUC2B* were accumulated more strongly in *cuc2b* than in the WT. Although amino acid substitution appears to have little effect on the protein stability (Supplementary Fig. S3), a potential impact on protein function and mutant phenotype cannot be excluded. The reduced expression of the other *CpCUC1* and *CpCUC2* genes (Fig. 5) in the same apical shoots suggests a competition between the different *CUC* genes for binding *miR164*, but clearly demonstrates a major function for *CpCUC2B* in the developmental functions defined by *cuc2b*. Despite this, given the functional redundancy of Arabidopsis *CUC* genes, with *cuc1* and *cuc2* single mutants having a nearly normal phenotype (Takada *et al.*, 2001), it is likely that the partial penetrance of *cuc2b* in different plants and flowers may depend on the combined effect of *CpCUC1* and *CpCUC2* genes, up-regulating *CpCUC2B*, but down-regulating *CpCUC2A* and *CpCUC1A* in overlapping tissues. Therefore, it cannot be ruled out that *CpCUC1A* and *CpCUC2A* can complement the defects produced by *cuc2b* in certain developmental processes.

### *CpCUC2B* has multiple functions in the formation of the axillary floral meristem and the development of the lateral organs

The phenotype of *cuc2b* has shown that *CpCUC2B* plays a relevant role in defining the boundaries of the floral organs and the leaf. The role of *CUC* genes in boundary specification and lateral organ separation is conserved in different plant species, including the dicots Petunia, Arabidopsis, tomato, and Medicago (Souer *et al.*, 1996; Aida *et al.*, 1997; Weir *et al.*, 2004; Berger *et al.*, 2009; Cheng *et al.*, 2012), and monocots such as rice and sugarcane (Chang *et al.*, 2021; Aslam *et al.*, 2022). For this reason, this family of genes are known as boundary genes. The inferred regulatory network of the *CpCUC2A/B* gene also supports the involvement of these genes in organ patterning and in reproductive development.

The loss of function of *cuc* mutations hinders the formation of boundaries in the floral meristem and the separation of floral organs, leading to flowers with fused and a reduced number of floral organs (Aida *et al.*, 1997; Vroemen *et al.*, 2003; Hibara *et al.*, 2006). During leaf development, loss-of-function mutations result in fused or a low number of leaflets of compound leaves and reduced serration of Arabidopsis simple leaves (Blein *et al.*, 2008; Berger *et al.*, 2009; Cheng *et al.*, 2012; Wang and Dane, 2013). In contrast, the squash *cuc2b* gain-of-function mutation leads to an increase in the number of sepals, petals, and stamens, and to a deeper and larger leaf serration, a phenotype also exhibited by overexpressed *CUC* mutants resulting from a defective *miR164* regulation in Arabidopsis (Laufs *et al.*, 2004; Baker *et al.*, 2005; Nikovics *et al.*, 2006) and the *miR164*-resistant variant of tomato *SlGOB* (Berger *et al.*, 2009).

In squash, solitary male or female flowers are formed on the axil of each leaf. The development of double male and female flowers in the squash *cuc2b* mutant therefore shows the crucial role of *CpCUC2B* in the formation of the floral meristem, a function that has not been previously reported for the *CUC* genes. However, the *cuc2b* mutation does not appear to be very severe since only 17.6 and 37.4 of the *cuc2b* nodes were occupied by double flowers, and the rest of the nodes develop single flowers. Double flowers are likely to be due to the formation of an accessory boundary in the floral meristem that eventually results in two separated or fused male or female flowers (Fig. 2). Division of the floral meristem must occur after the initiation of the primordia of sepals and petals, because in these double flowers the number of sepals and petals is reduced, while in single flowers the number of sepals and petals increases compared with the WT (Table 2). Although floral meristems do not appear to be affected by *cuc* mutations in Arabidopsis, the *cuc2b* double axillary flowers resemble those of the axillary vegetative meristem in Arabidopsis *cuc* gain-of-function mutants, which develop accessory vegetative buds in leaf axils (Raman *et al.*, 2008). Therefore, it is likely that *CUC* genes are required for the formation of axillary meristems, be they axillary flowers or secondary axillary shoots.

### *CpCUC2B* controls squash sex determination by arresting the development of carpels and suppressing the arrest of stamens

The role of *CpCUC2B* in sex determination is more specific for squash and perhaps other cucurbits. The formation of female and male unisexual flowers in monoecious and dioecious plants results from the arrest of either stamens or carpels in a floral meristem that is initiated as hermaphrodite. In cucurbits, ethylene is the hormone that determines sex, arresting stamen development and promoting carpel development for the determination of female flowers. A reduction in ethylene at very early stages of flower development leads to the activation of the WIP1 zinc finger transcription factor, which arrests the development of carpels during the determination of male flowers (Martin *et al.*, 2009; Hu *et al.*, 2017; Zhang *et al.*, 2020). The role of *CpCUC2B* is similar to that described for *WIP1* and opposite to that of ethylene. The increased number of male flowers in *cuc2b* plants and the reduced number of carpels in *cuc2b* pistillate flowers clearly show that the gene is required to arrest carpel development for the determination of male flowers. Comparison of the transcriptomes of male and hermaphrodite flowers in the androdioecious tree *Tapiscia sinensis* also suggested that *CUC* genes may promote the formation of male flowers by suppressing carpel development in hermaphrodite floral meristems (Xin *et al.*, 2019). Furthermore, the fact that 2.4% of the *cuc2b* pistillate flowers were classified as bisexual, developing an ovary and stamens, but without a pistil or stigma, also indicates that *CpCUC2B* is also able to promote the development of stamens in a floral meristem that was already determined as female. Down-regulation of *CpACS27A* in *cuc2b* female apical shoots also supports the conclusion that *CpCUC2B* suppresses stamen arrest in female flowers. This function was described for *WIP1* in melon and cucumber, which is negatively regulated by ethylene biosynthesis genes such as *ACS11* and *ACO2* at very early stages of flower development (Boualem *et al.*, 2015; Chen *et al.*, 2016), but later suppresses the activation of ethylene biosynthesis genes that are involved in stamen abortion (*CmACS7*, *CsACS2*, *CitACS4*, and *CpACS2*7 orthologs), which leads to the floral meristem being determined as a male flower (Martínez and Jamilena, 2021). The squash *CpWIP1B* is highly expressed in the apical shoots of plants at the male phase of development (Fig. 6E), suggesting that it cooperates with *CpCUC2B*, and perhaps other *CUC*-like genes, in the specification of male flowers. The function of the *CUC* genes, as repressors of growth and organ outgrowth to define boundaries and organ separation (Aida and Tasaka, 2006), could have been exploited to promote carpel abortion during the evolutionary emergence of unisexual flowers in monoecious cucurbits. The WIP1 transcription factor has also been described as an inhibitor of growth and development by activating genes involved in programmed cell death and repressing genes involved in meristem growth (Roldan *et al.*, 2020). In the few pistillate flowers of *cuc2b*, the ovary phenotype is similar to that of Arabidopsis mutants that express *miR164*-resistant forms of *CUC1* and *CUC2* (Sieber *et al.*, 2007; Kamiuchi *et al.*, 2014), and indicates that *CpCUC2B* conserves its role in placenta and ovule development.

**Fig. 7.**
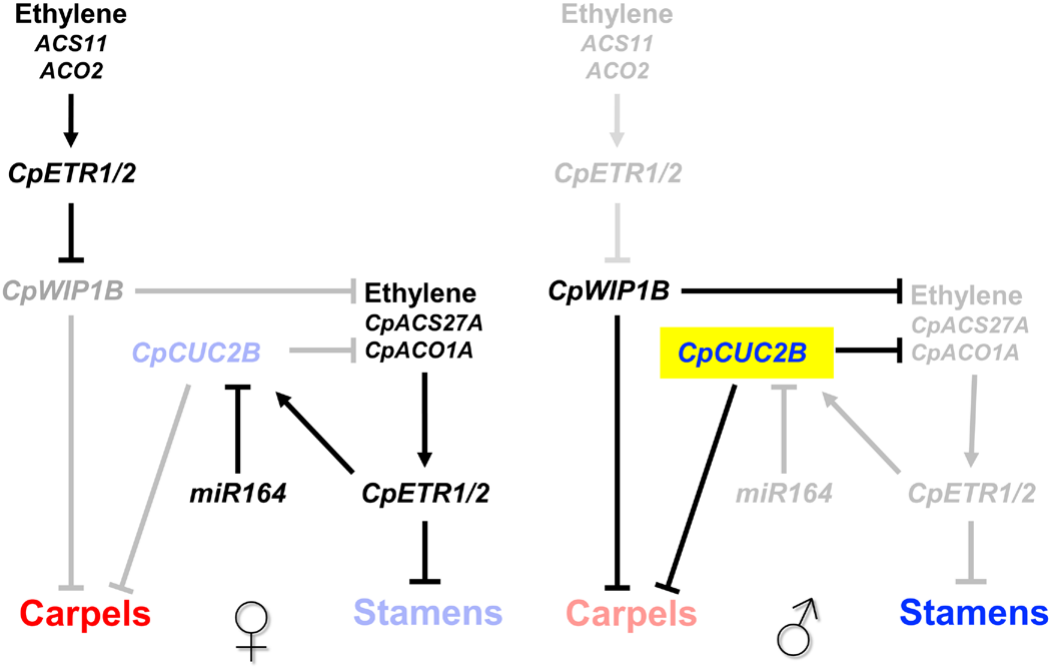
Role of *CpCUC2B* and *miR164* in the gene network model regulating the arrest of stamens and carpels for female and male flower determination, respectively. The function of *CpCUC2B* in the arrest of carpel development for the development of male flowers is independent of ethylene produced by *ACS11* and *ACO2*, and of the transcription factor *CpWIP1B*. However, *CpCUC2B* inhibits the ethylene produced by CpACS27A and CpACO1A, the enzymes involved in the arrest of stamen during the development of female flowers. Genes that are active in male or female flower development are highlighted in black or a more intense color, while those that are repressed are in gray or a paler color.

How is *CpCUC2B* integrated in the genetic framework that controls cucurbit sex determination? Most of the known cucurbit sex-determining genes are ethylene biosynthesis and signaling genes. So, we studied the genetic interactions between *cuc2b* and the already known mutations in ethylene biosynthesis and signaling pathways. It was known that squash ethylene receptors CpETR1A, CpETR1B, and CpETR2B perceive not only the early ethylene produced by ACS11 and ACO2, promoting in this way development of carpels by repressing WIP1, but also the late ethylene that is produced by the enzymes CpACS27A and CpACO1A and that triggers stamen abortion. The phenotypes of single and double mutants, and the expression of *CpCUC2B*, *CpWIP1B*, and ethylene sex-determining genes in the ethylene and *cuc2b* mutants, permitted the development of the model shown in Fig. 7. The ethylene-insensitive mutations (*etr1b*, *etr1a*, *etr1a-1*, and *etr2b*) convert female flowers into male or hermaphrodite flowers, thus generating a sexual phenotype that goes from andromonoecy to androecy, depending on the severity of each mutation and the level of ethylene insensitivity of each mutant (García *et al.*, 2020a, b). However, the *aco1a* mutation only converts female flowers into hermaphrodite flowers but does not produce any change in the number of male and pistillate flowers per plant (Cebrián *et al.*, 2022). The sex phenotype of the double mutant *cuc2b/aco1a* demonstrated that the *cuc2b* mutation is epistatic over *aco1a* and, given that the *CpACS27A* gene is significantly down-regulated in *cuc2b*, it is likely that *CpCUC2B* promotes stamen development by suppressing CpACS27A, the key enzyme involved in late ethylene production and stamen abortion in female flowers (Fig. 7). Down-regulation of *CpCUC2A* and *CpCUC2B* in the apical shoots of *aco1a* may indicate that this late ethylene may finally activate the *CUC* genes required for the specification and separation of female floral organs (Fig. 7). This conclusion is also supported by the reduced boundary-specific *cuc2b* phenotypes in the female flowers compared with male flowers of the double mutant *cuc2b*/*aco1a* (Table 5).

On the other hand, the androecious phenotype of the double mutant *cuc2b/etr2b*, where all female flowers were converted into male flowers, demonstrated a synergistic effect of the *cuc2b* and *etr2b* mutations on male flower production. The role of *CpCUC2B* in carpel abortion during the determination of a male flower is therefore independent of ethylene (Fig. 7), a regulation that differs from that of *WIP1*, whose expression is negatively regulated by *ACS11* and *ACO2* in female floral meristems of melon and cucumber (Boualem *et al.*, 2015; Chen *et al.*, 2016). The fact that the study of gene expression did not show transcriptional regulation between the *CpCUC* and *CpETR* genes (Fig. 6) also supports the independent action of *CpCUC2B* and ethylene in the suppression or promotion of carpel development in squash flowers. A more in-depth study would be required to determine whether the role of ethylene in the promotion of carpel development could be mediated by the down-regulation of *CpWIP1B* in squash. The present data, however, indicate that *CpWIP1B* may be involved in male flower specification, since the gene is highly expressed in the apical shoots of plants at the early male phase of development (Fig. 6E).

## Supplementary data

The following supplementary data are available at *JXB* online.

Dataset S1. Validation of the *cuc2b* mutation.

Dataset S2. Regulatory network of *CUC* genes inferred by the ARACNE algorithm.

Table S1. List of the *CUC* genes and proteins used in alignments and phylogenetic analysis.

Table S2. Primers used for gene expression and mRNA degradation analysis by qPCR.

Fig. S1. Effects of the *cuc2b* mutation on vegetative development.

Fig. S2. Multiple alignment of CpCUC2 amino acid sequences with homologous CUC2 proteins from diverse species.

Fig. S3. Effect of *cuc2b* mutation on protein stability.

Fig. S4. Multiple alignment of *CpmiR164* mature sequence with homologous *miR164* from diverse plant species.

Fig. S5. Loci encoding *miR164* in *C. pepo*.

Fig. S6. qRT-PCR of the *CpCUC2B* gene.

Fig. S7. *CUC* gene regulation network.

Fig. S8. Phenotypes of single and double mutants of *etr2b*, *aco1a*, and *cuc2b*.

erad486_suppl_Supplementary_Tables_S1-S2_Figures_S1-S8

erad486_suppl_Supplementary_Datasets_S1-S2

## Data Availability

All relevant data can be found within the manuscript and its supporting materials. All the raw reads generated in this study have been deposited in the public database of the National Center of Biotechnology under BioProject PRJNA1042934.
