# Supplementary material for: An miR164-resistant mutation in the transcription factor gene CpCUC2B enhances carpel arrest and ectopic boundary specification in Cucurbita pepo flower development
Source: J Exp Bot. 2023 Dec 8;75(7):1948–66. doi: 10.1093/jxb/erad486 (PMC10967242; doi:10.1093/jxb/erad486)
Supplement: erad486_suppl_Supplementary_Tables_S1-S2_Figures_S1-S8 [file erad486_suppl_supplementary_tables_s1-s2_figures_s1-s8.pdf]

**Supplementary Table S1.** List of the CUC genes and proteins used in alignments and phylogenetic analysis.

| Species | GeneID | Gene name |
|---|---|---|
| ***Cucurbita pepo*** | 111810386 | *CpCUC1A* |
| | 111803201 | *CpCUC1B* |
| | 111789288 | *CpCUC2A* |
| | 111797633 | *CpCUC2B* |
| | 111805279 | *CpCUC3A* |
| | 111789055 | *CpCUC3B1* |
| | 111799080 | *CpCUC3B2* |
| ***Cucurbita maxima*** | 111476901 | *CmaxCUC2* |
| ***Cucurbita moschata*** | 111442545 | *CmosCUC2* |
| ***Cucumis sativus*** | 101205357 | *CsCUC2* |
| ***Cucumis melo*** | 103501784 | *CmCUC2* |
| ***Arabidopsis thaliana*** | 820748 | *AtCUC1* |
| | 835478 | *AtCUC2* |
| | 843975 | *AtCUC3* |
| ***Solanum lycopersicum*** | 100301934 | *SlGOB* |
| ***Capsicum annuum*** | 107877396 | *CaCUC2* |
| ***Prunus persica*** | 18779165 | *PpCUC2* |
| ***Vitis vinifera*** | 100246914 | *VvCUC2* |
| ***Medicago truncatula*** | 25487304 | *MtCUC2* |
| ***Carica papaya*** | 110808876 | *CpaCUC2* |
| ***Malus domestica*** | 103419033 | *MdCUC2* |

**Supplementary Table S2:** Primers used for gene expression and mRNA degradation analysis by qPCR.

| Gene | Primer | Sequence |
|---|---|---|
| ***CpACO1A*** | F | CATAGAGTGATGACTCAGACAAGC |
| | R | CCATTGGACCCAAATTAGCA |
| ***CpACS11A*** | F | CGTCGTCTTAAGGCCTTTG |
| | R | GGTGTACCTAATTTAACGCAAC |
| ***CpACO2B*** | F | GGAGGGAGAGGAAGATAAGG |
| | R | TGGGTTATTGGAAAATGGAG |
| ***CpWIP1B*** | F | TCCCTACTCCATGCTTCAC |
| | R | TCCTCCTCATTCACAACAAC |
| ***CpACS27A*** | F | CCAATACGGACGGTGAA |
| | R | GGAGAAGCTGAAGAAGGAAG |
| ***CpCUC1A_mir164*** | F | GGATCCCGAACCGAGTTCCT |
| | R | GACCGAGGGGGAGAGATCAG |
| ***CpCUC1B_mir164*** | F | GCCGACACCAGTATCAGCAC |
| | R | AGACGGACAGATTAGGACCGC |
| ***CpCUC2A_mir164*** | F | GCCACTTCTCGACTCGTCACTT |
| | R | GTTTCGGTGGAAGCGGGTAG |
| ***CpCUC2B_mir164*** | F | GCCGCTGCTAGATTCGTCTT |
| | R | GAGGGGGAGTGGCAAAATCG |
| ***CpCUC2B_3'*** | F | TACGATTTTGCCACTCCCCC |
| | R | AGGACATGTTGCCATGGTGG |
| ***CpEF1α*** | F | CGTCAAGAAGAAATAAGCCA |
| | R | CTACTACGAGAGAGAGAGCCG |
| ***CpACT*** | F | CCTCTCAATCCCAAAGCTA |
| | R | CGGCCTGGATAGCAACATA |

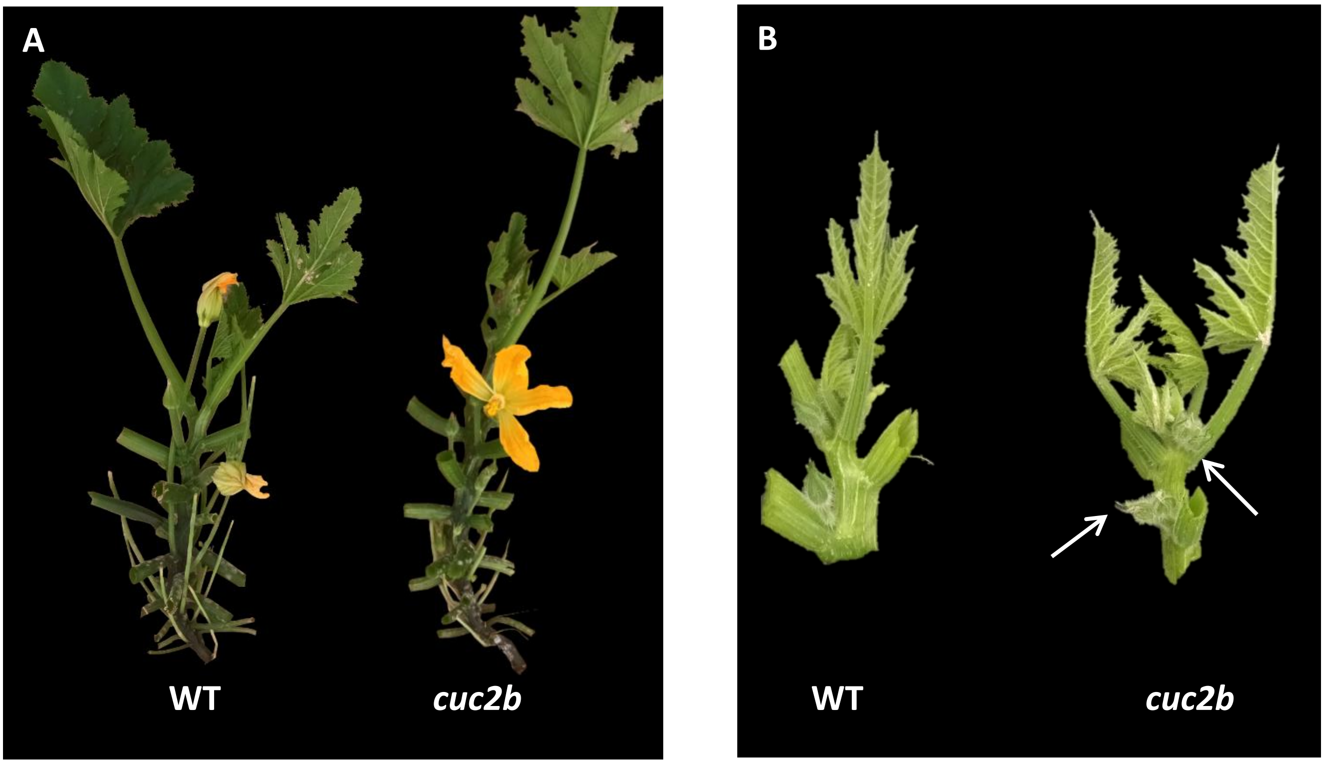


**Supplementary Figure S1.** Effects of the *cuc2b* mutation on vegetative development. **(A)** WT and *cuc2b* complete plant with removed old leaves and flowers, showing the main stem. The absence of lateral branches and unaltered apical dominance is observed. **(B)** Detail of the apical shoot of WT and *cuc2b* plants. White arrows indicate double flowers.

```
CpCUC2B       MDVSSFSRYDSAAV-DHPHPHHLPPGFRFHPTDEELITYYLLKKVLDSSFSGRAIAEVDL   59
CpCUC2A       MDVSPFSHYDD--------SHHLPPGFRFHPTDEELITYYLLKKVLDSTFTGRAIAEVDL   52
CpCUC1B       ------MDHGGSGGGHQSSDGFLPPGFRFHPTDEELIAHYLLKKVLDSHFSSPAITHVDL   54
CpCUC1A       ---------MDGSVHRSSNNGYLAPGFRFHPTDEELIAHYLLKKVLDSHFSSPAIAYVDL   51
CmaxCUC2      MDVSSFSRYDSAAA-ADHHPHHLPPGFRFHPTDEELITYYLLKKVIDSSFSGRAIAEVDL   59
CmosCUC2      MDVSSFSRHDSAAAAADHHPHHLPPGFRFHPTDEELITYYLLKKVLDSSFSGRAIAEVDL   60
CsCUC2        MDVSSFSHYDSAA--SDNSNHHLPPGFRFHPTDEELITYYLLKKVLDTNFTGRAIAEVDL   58
CmCUC2        MDVSSFSYYDSA---ADNTNHHLPPGFRFHPTDEELITYYLLKKVLDTNFTGRAIAEVDL   57
CaCUC2        --------MENYQMHFDCGDPHLPPGFRFHPTDEELITYYLLKKVLDSNFTARAIAEVDL   52
AtCUC2        MDIPYYHY------DHGGDSQYLPPGFRFHPTDEELITHYLLRKVLDGCFSSRAIAEVDL   54
CpaCUC2       METYIH-------HHFDNCDAHLPPGFRFHPTDEELITYYLLKKVLDSSFTGRAIAEVDL   53
MtCUC2        MESFFHHHHNMNNNSNNNSDAHLPPGFRFHPTDEELITFYLLKKVLDTTFTARAIAEVDL   60
PpCUC2        MDIMSFTGH-----FDHGSDTHLPPGFRFHPTDEELITYYLLKKVLDSSFTGRAIAEVDL   55
VvCUC2        --MDAY-------HHFDNSDAHLPPGFRFHPTDEELITYYLIKKVLDSNFTGRAIAEVDL   51
MdCUC2        MDIMSFSTH-----FDHN--THLPPGFRFHPTDEELITYYLLKKVLDSSFTGRAIAEVDL   53
                                   .* *************:.**::**:*  *:. **: ***

CpCUC2B       NKCEPWELPEKAKMGEKEWYFFSLRDRKYPTGLRTNRATEAGYWKATGKDREIYSVKTCS   119
CpCUC2A       NKCEPWELPEKAKMGEKEWYFFSLRDRKYPTGLRTNRATEAGYWKATGKDREIYSAKTYS   112
CpCUC1B       NKSEPWDLPEKALMGEKEWYFFSLRDRKYPTGLRTNRATEAGYWKATGKDREIMSSKTNS   114
CpCUC1A       NKSEPWDLPEKAVMGEKEWYFFSLRDRKYPTGLRTNRATEAGYWKATGKDREITSSKTNS   111
CmaxCUC2      NKCEPWELPEKAKMGEKEWYFFSLRDRKYPTGLRTNRATEAGYWKATGKDREIYSVKTCS   119
CmosCUC2      NKCEPWELPEKAKMGEKEWYFFSLRDRKYPTGLRTNRATEAGYWKATGKDREIYSVKTCS   120
CsCUC2        NKCEPWELPEKAKMGEKEWYFFSLRDRKYPTGLRTNRATEAGYWKATGKDREIYSVKTCS   118
CmCUC2        NKCEPWELPEKAKMGEKEWYFFSLRDRKYPTGLRTNRATEAGYWKATGKDREIYSVKTCS   117
CaCUC2        NKCEPWELPGKAKMGEKEWYFFSLRDRKYPTGLRTNRATEAGYWKATGKDREIFSSKTCA   112
AtCUC2        NKCEPWQLPGRAKMGEKEWYFFSLRDRKYPTGLRTNRATEAGYWKATGKDREIFSSKTCA   114
CpaCUC2       NKCEPWELPEKAKMGEKEWYFFSLRDRKYPTGLRTNRATEAGYWKATGKDREIYSSKTCA   113
MtCUC2        NKCEPWELPEKAKMGEKEWYFFSLRDRKYPTGLRTNRATEAGYWKATGKDREIYSSKTYS   120
PpCUC2        NKCEPWELPDKAKMGEKEWYFFSLRDRKYPTGLRTNRATEAGYWKATGKDREIYSSKTCA   115
VvCUC2        NKCEPWELPEKAKMGEKEWYFFSLRDRKYPTGLRTNRATEAGYWKATGKDREIYSSKTCS   111
MdCUC2        NKCEPWELPDKAKMGEKEWYFFSLRDRKYPTGLRTNRATEAGYWKATGKDREIYSSKTCA   113
              **.***:** :* **************************************** * ** :

CpCUC2B       LVGMKKTLVFYRGRAPKGEKSNWVMHEYRLEGKFAYHYLST-NSKDEWVISRVFQKSGTG   178
CpCUC2A       LVGMKKTLVFYRGRAPKGEKSNWVMHEYRLEGNFAYHYLSR-TSKDEWVISRVFQKSGAG   171
CpCUC1B       LIGMKKTLVFYRGRAPKGQKTNWVMHEFRLAGKFAYHFLSADSSKDEWVISKVFKKTGAA   174
CpCUC1A       VVGMKKTLVFYTGRAPKGQKTHWVMHEFRLAGKFAYHFLSAASSKDEWVISRVFKKTSAA   171
CmaxCUC2      LVGMKKTLVFYRGRAPKGEKSNWVMHEYRLEGKFAYHYLST-NSKDEWVISRVFQXSGTG   178
CmosCUC2      LVGMKKTLVFYRGRAPKGEKSNWVMHEYRLEGKFAYHYLST-NSKDEWVISRVFQKSGTG   179
CsCUC2        LVGMKKTLVFYRGRAPKGEKSNWVMHEYRLEGKFAYHYLSG-SSKDEWVISRVFQKSGSG   177
CmCUC2        LVGMKKTLVFYRGRAPKGEKSNWVMHEYRLEGKFAYHYLSG-SSKDEWVISRVFQKSGSG   176
CaCUC2        LVGMKKTLVFYRGRAPKGEKSNWVMHEYRLDGKFAYHYISR-SSKDEWVISRVFQKSATA   171
AtCUC2        LVGMKKTLVFYKGRAPKGEKSNWVMHEYRLEGKFSYHFISR-SSKDEWVISRVFQKTTLA   173
CpaCUC2       LVGMKKTLVFYRGRAPKGEKSNWVMHEYRLEGKFAYHYLSR-SSKDEWVISRVFQKSGAG   172
MtCUC2        LVGMKKTLVFYRGRAPKGEKSNWVMHEYRLEGKFAYHFLSR-NSKDEWVISRVFQKSNTG   179
PpCUC2        LVGMKKTLVFYRGRAPKGEKSNWVMHEYRLEGKFAYHYLSR-SSKDEWVISRVFQKSSGS   174
VvCUC2        LVGMKKTLVFYRGRAPKGEKSNWVMHEYRLEGKFAYHYLSR-SSKDEWVISRVFQKSGSS   170
MdCUC2        LVGMKKTLVFYRGRAPKGEKSNWVMHEYRLEGKFAYHYLSR-SSKDEWVISRVFQKSSGS   172
              ::********* ******:*::*****:** *:*:**::*  .********:**: :  .

CpCUC2B       VG--------GG---VDGGGG-SRKPRFIP-----GTVSVYPEPSSPSSV-SLPPLLDSS   220
CpCUC2A       GG--------GGGATSSNGGG-SKKARFVP-----GSVNVYPEPSSPSSV-SLPPLLDSS   216
CpCUC1B       MHG----------------GSSTRK------------SRLDPEPSSSSSV-SLPPLLDSS   205
CpCUC1A       MHG----------------GVM-KK------------TRRDPEPTSPSSV-SLPPLLDSS   201
CmaxCUC2      VG--------GG---VDGGGG-SRKPRFIP-----GTVSVYPEPSSPSSV-SLPPLLDSS   220
CmosCUC2      VG--------GG---VDGGGG-SRKPRFIP-----GTVSVYPEPGSPSSV-SLPPLLDSS   221
CsCUC2        ----------CGGATSSNGGS-SKKARFIP-----GSINVYPEPSSPSSV-SLPPLLDSS   220
CmCUC2        ----------CGGATSSNGGG-SKKARFIP-----GSINVHPEPSSPSSV-SLPPLLDSS   219
CaCUC2        IT--------GTA--TSNGGK-KRP--------SSSINNMYQEVSSPSSVSHLPPLLDSS   212
AtCUC2        STGAVGGEGGGATVSVSSGTGPSKKTKVP----STVSRNYQEQPSSPSSV-SLPPLLDPT   228
CpaCUC2       SC--------G--------GG-GKKTKMP------PSINLYQEPSSPSSV-SLPPLLDPT   208
MtCUC2        NG--------STTVTVATTSG-AKKTKIASNSASNNSMSLCPEPSSPSSV-YLPPLLDSS   229
PpCUC2        T---------------TNGPG-SKKTRMSN--GSTSSISLYPEPSSPSSV-SLPPLLDSS   215
VvCUC2        GG--------GGA------TG-GKKARLS------STVNLYPEVSSPSSA-SLPPLLDVS   208
MdCUC2        A---------------ANGPG-SKKTRMGN--GSTSSVSLYPEPSSPSSV-SLPPLLDSS   213
                                     :                  :  * **.  ****** :
```

```
CpCUC2B          LYSAPVAATAVSGN-ITDRDSCSYNS-----------P--REHVSCFST-TAGGSFNIPS  265
CpCUC2A          LYSAPATISTASGN-IPDRDSRSYNS-----------PAAREHVSCFST-TAGGSLNIPN  263
CpCUC1B          A------------------------D----------PSEHELHVTCFSTNNVNY------  225
CpCUC1A          PYLSAAADTSISTSAFANREGSSLDD----------QREHELHVTCFSTNNLSYPAAS--  249
CmaxCUC2         LYSAPVAATAVSGN-ITDRESFSYNS-----------P--REHVSCFST-TTGGSFNIPS  265
CmosCUC2         LYSAPVAATAVSGN-ITDRDSCSYNS-----------P--REHVSCFST-TTGGSFNIPS  266
CsCUC2           LYSAPATGTAASGN-ITDRDSCSYNS-----------PTAREHVSCFSTTTAGGSFNMPN  268
CmCUC2           LYSAPATGTAASGN-ITDRDSCSYNS-----------PTAREHVSCFSTTTAGGSFNIPS  267
CaCUC2           PYSTTATSSSVVIS---A-----RDH-----------SFKKEHVSCFSTTATNSATIDPH  253
AtCUC2           TTLGY------------TDSSCSYDSRSTN--TTVTASAITEHVSCFSTVPTTTTALGLD  274
CpaCUC2          TASTT-------AA-ATSLDSCSYDGHSFSTSAAAAAAASSEHVSCFSTIAAAAAAAGSS  260
MtCUC2           PYTAASTASFNGGQ-MCNYNS-SNNN-----------TDLKEHVSCFSTTSTTSNVVSVH  276
PpCUC2           PYQNMN-----TAG-LTDRDSCSYDS-----------PIPKEHVSCFSTNP-NSGFNLSS  257
VvCUC2           PYAGTSAA----AA-VNDRESCSYDGGESSNN----SNARDQHVPWFSTIAAAAAAAAAN  259
MdCUC2           PYQPIS-----AAG-----DGCSYDS-----------PIPREHVSCFSTNPSNNGFNLPA  252
                                                           **  ***

CpCUC2B          --Y--------DFAT-PPPLLAAVDPSPRFHRNMGLSAFPSLRSLQENLQ-LPFFYSPVN  313
CpCUC2A          --Y--------DFAP-LPPLA-AADPSTRFHRNIGLSAFPSLRSLQENLQ-LPFFYSPAN  310
CpCUC1B          --------------------------PAGGGSDLSPSVFPNLLSFQENLQ-APFFSP-VG  257
CpCUC1A          ------------F-------------PAGGGPNLSVSAFPNLRSIQENLQ-APFFSP-VS  282
CmaxCUC2         --Y--------DFAT-PPPLLAAVDPSPRFHRNMGLSAFPSLRSLQENLQ-LPFFYSPVN  313
CmosCUC2         --Y--------DFAT-PPPLLAAVDPSPRFHRNMGLSAFPSLRSLQENLQ-LPFFYSPVN  314
CsCUC2           --Y--------DFAT-PSPLL-AADPSPRFHRNIGLSAFPSLRSLQENLQ-LPFFYSPVN  315
CmCUC2           --Y--------DFAT-PSPLL-AADPSPRFHRNIGLSAFPSLRSLQENLQ-LPFFYSPVN  314
CaCUC2           --S--------VFDI-SNTLH-----ALPFTHYGRNSTFPSLTSLHENLQ-LPLFSGGA-  295
AtCUC2           VN---------SFSRLPPPLGFDFDPFPRFVSR-NVSTQSNFRSFQENFNQFPYFGSSSA  324
CpaCUC2          ASSTFDFGPPPQLPP-QPQMYAGYDSFTRFPRSVGLSGFPSLRSLQENLQ-LPFFFSSST  318
MtCUC2           --NNFNSNSNGSFDLVSPSMNATMDPFARFQRNVGVSAFPSLRSLQDNLQ-LPFFFSSAA  333
PpCUC2           --S--------CFDL-AH------AQPPPQPSFGGVSAFPSLRSLQENLQ-LPFFFSPMS  299
VvCUC2           SFNAHH--QPPPFDL-APPSIIGSIDPSRFPRNGAVPAFPNLRSLQENLH-LPFFFSQVA  315
MdCUC2           --S--------CFDL-AH------PPPPQPPNFGGVSAFPSLRSLQENLQ-LPFFFSPMP  294
                                                         .: *:::*::  * *

CpCUC2B          LPSPPPPPLHHGNMSSSADVVACNSAGNWG----VSD---------EPRSMNSTEL-DCM  359
CpCUC2A          LPSTTAVH--HG-NISSADIGACSSAGNWA----VSD---------ETRFMNSTEL-DCM  353
CpCUC1B          PPLVHLLT-------DAAELVGCG---DGPAPE-------------DSRLVGSADMAAYM  294
CpCUC1A          PPLVHLLS-------GAADVDGCG---GAPSPE-------------DSRTIGSADLGAYM  319
CmaxCUC2         LPSPPPPPLHHGNIPSSTDVVACNSAGNWA----VSD---------EPRSMNSTEL-DCM  359
CmosCUC2         LPSPPPPPLHHGNMSSSADVAACNSAGNWA----VSD---------EPRSMNSTEL-DCM  360
CsCUC2           LCSPPVP---VL-NNGN-DVAGCSSGGNWG----VSD---------EPKVMNSTEL-DCM  356
CmCUC2           LCSPPVP---AL-NNGNNDVAACSSGGNWG----VAD---------EPKVMSSTEL-DCM  356
CaCUC2           ---------------SAMHGGFSNSIGNWSVPE--------------TQKVEQSEL-DCM  325
AtCUC2           STMTSSVN------LPSFQGGGGVSGVNYWLPATAEE-------NESKVGVLHAGL-DCI  370
CpaCUC2          SSPSASIP-------PPLFQGGGGAAFSWPFM--PDE----GG---GKLCMGASEL-DCM  361
MtCUC2           AAQPFPGGGD--------------ILGGWGMPSEEQRVVVDSGGGLNMGGLGSSEL-DCM  378
PpCUC2           GHQPVHVGSSGGGGGSTIDLGGLSSAGSWPTPPPHQE---------EPRTVGPTEL-DCM  349
VvCUC2           PPIPSSGD-------PSTEMGITNSAGNWPAPENQKM----DN---GRLPMGATEL-DCM  360
MdCUC2           AHPPVHVGSSVAGGGSSVDFT-----GNWP---PVSE---------EARAVGPTEL-DCM  336
                                            .                      :  : :   :

CpCUC2B          WSY------       362
CpCUC2A          WSY------       356
CpCUC1B          WTF------       297
CpCUC1A          WTF------       322
CmaxCUC2         WSY------       362
CmosCUC2         WSY------       363
CsCUC2           WSY------       359
CmCUC2           WSY------       359
CaCUC2           WTY------       328
AtCUC2           WNY------       373
CpaCUC2          WSF------       364
MtCUC2           WNY------       381
PpCUC2           WTYYKTECL       358
VvCUC2           WSY------       363
MdCUC2           WNYYKTECL       345
                 *.:
```

NAC domain

miR164 binding site

*cuc2b* mutation effect: S251F

**Supplementary Figure S2**: Multiple alignment of CpCUC2 amino acid sequences with homologous CUC2 proteins from diverse species. Blue boxes indicate the conserved NAC domain, yellow box the amino acid sequence of the miR164 binding site. The amino acid transition cause by the *cuc2b* mutation is highlighted in red.

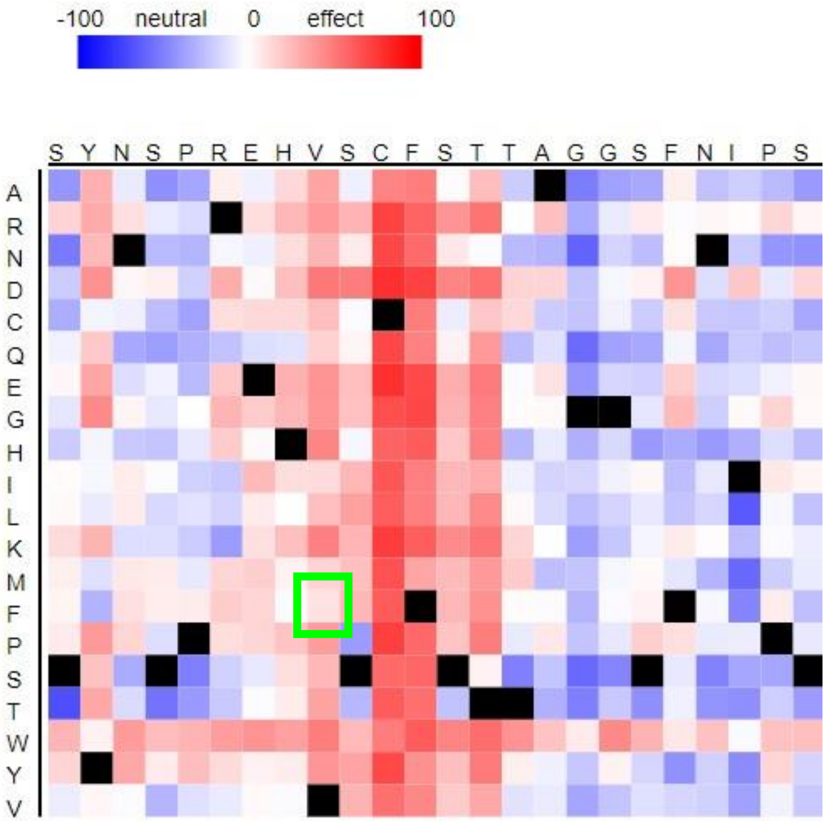


**Supplementary Figure S3**: Protein stability analysis of the S251F change in CUC2 protein. Heat map obtained by SNAP2 tool suggest a reduced effect of the mutation in the protein function.

```
Consensus      UGGAGAAGCAGGGCACGUGCA
CpmiR164       UGGAGAAGCAGGGCACGUGCA        21
CmmiR164a      UGGAGAAGCAGGGCACGUGCU        21
CmmiR164b      UGGAGAGGCAGGGCACAUGCU        21
CmmiR164c      UGGAGAAGCAGGGCACGUGCA        21
CmmiR164d      UGGAGAAGCAGGGCACGUGCA        21
AtmiR164a      UGGAGAAGCAGGGCACGUGCA        21
AtmiR164b      UGGAGAAGCAGGGCACGUGCA        21
AtmiR164c      UGGAGAAGCAGGGCACGUGCG        21
CpamiR164a     UGGAGAAGCAGGGCACGUGCA        21
CpamiR164b     UGGAGAAGCAGGGCACGUGCA        21
CpamiR164c     UGGAGAAGCAGGGCACGUGCA        21
CpamiR164d     UGGAGAAGGGGAGCACGUGCA        21
CpamiR164e     UGGAGAAGGGGAGCACGUGCA        21
MtmiR164a      UGGAGAAGCAGGGCACGUGCA        21
MtmiR164b      UGGAGAAGCAGGGCACGUGCA        21
MtmiR164c      UGGAGAAGCAGGGCACGUGCA        21
MtmiR164d      UGGAGAAGCAGGGCACAUGCU        21
PpmiR164a      UGGAGAAGCAGGGCACGUGCA        21
PpmiR164b      UGGAGAAGCAGGGCACGUGCA        21
PpmiR164c      UGGAGAAGCAGGGCACGUGCA        21
PpmiR164d      UGGAGAAGCAGGGCACAUGCU        21
VvmiR164a      UGGAGAAGCAGGGCACGUGCA        21
VvmiR164b      UGGAGAAGCAGGGCACAUGCU        21
VvmiR164c      UGGAGAAGCAGGGCACGUGCA        21
VvmiR164d      UGGAGAAGCAGGGCACGUGCA        21
SlmiR164a      UGGAGAAGCAGGGCACGUGCA        21
SlmiR164b      UGGAGAAGCAGGGCACGUGCA        21
               ****** *  * **** ***
```

**Supplementary Figure S4**: Multiple alignment of *CpmiR164* mature sequence with homologous *miR164* from diverse plant species.

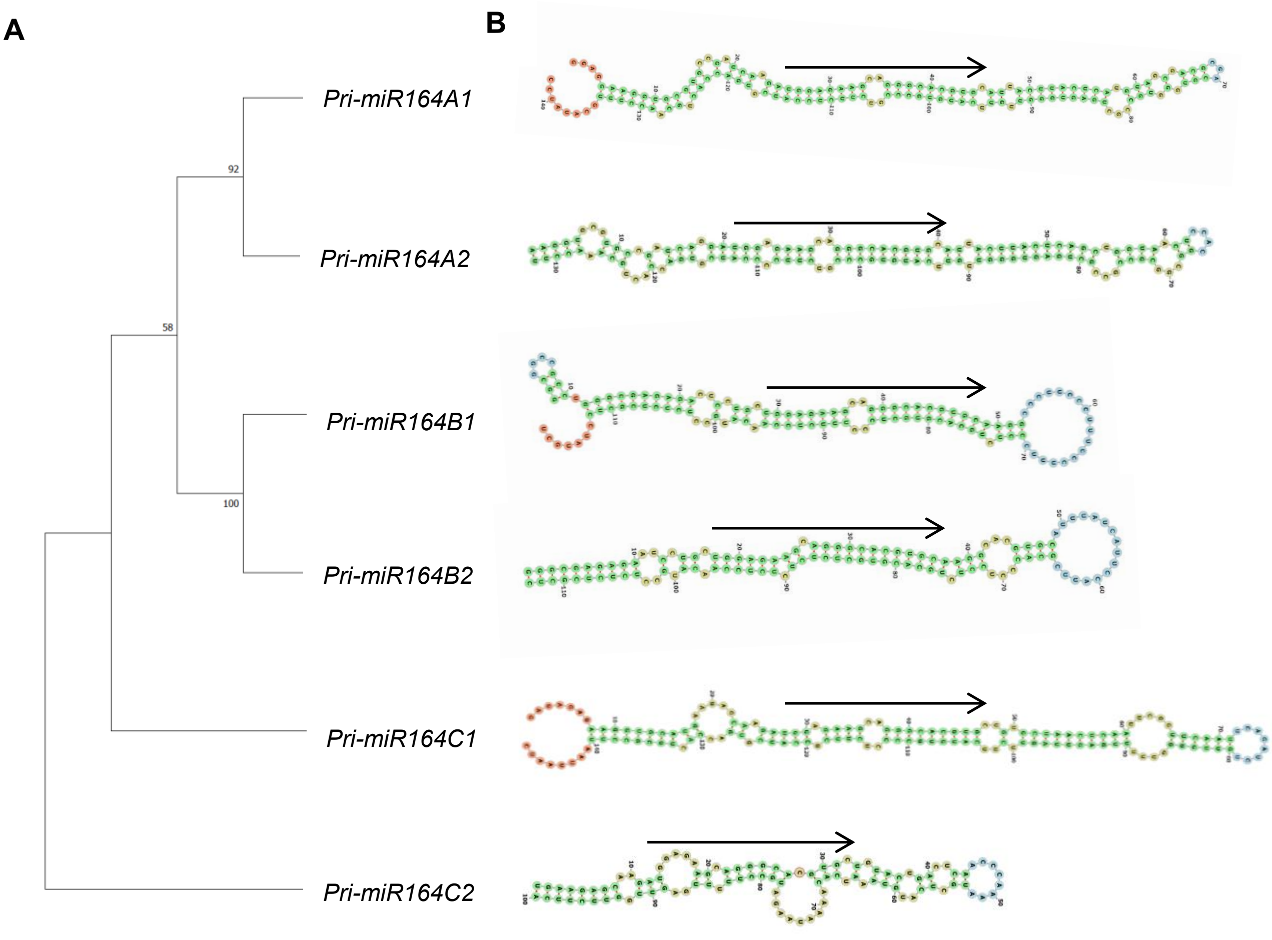


C

```
MIR164B1      GGCGGCCGCCUGGGCGAGAGACUCCUGCUGGAGAAGCAGGGCACGUGCAAGGCCCUUCCC  60
MIR164B2      -----------GGGCGAGAGAAUCCUGCUGGAGAAGCAGGGCACGUGCAAGGCACGUGCA  49
MIR164C2      ----------------UGAAGAGCAAGAUGGAGAAGCAGGGCACGUGCUGUACUGACUCA  44
MIR164C1      -GAGAGAGAAGGUGCAUGAAGAGCAAGAUGGAGAAGCAGGGCACGUGCUGUGCUAACUCA  59
MIR164A1      ---GGAGGAAGGUGCGUGCCGAUCAAGAUGGAGAAGCAGGGCACGUGCAUUACUUACUCA  57
MIR164A2      --------AAGGUGCGUGCCGAUCAGGAUGGAGAAGCAGGGCACGUGCAUUACUUACUCA  52
                               *     *  * ********************    *     *

MIR164B1      U--------------UUCCCUUUC-------------------------CCUCUGCACGU  81
MIR164B2      U--------------UUAUCAUUCCA-------------UUCGCACCCUCCUCUGCACGU  82
MIR164C2      ACCCAAAAUGCUCAUGUAAAUCACAAAAUAAGA-UGCCUUUUUGAGUUGGUUCUUCA---  100
MIR164C1      AUUCGG---------UUCAAUU--CAGAUCUGUUGGGGUUUUUGAGUUAGUUCUUCAUGU  108
MIR164A1      UGUAGC---------G--ACGC--GACGUG-GG---UGCCGCUGAGUGGGUUGUUCAUGU  100
MIR164A2      GCUGGU---------A--ACUC--CACGGC-GGCGCCGCCGCUGAGUGGGUUGUUCAUGU  98
                                                                 * * **

MIR164B1      GCUCCCUUUCUCCAACAUGCCUCUCUCGCUCCUAUGCU-----                   119
MIR164B2      GCUCUCUCUCUCCAACAUGCCUCUCUCGCUC------------                   113
MIR164C2      -------------------------------------------                   100
MIR164C1      GCCCCUCUUCGCCAUCGUGACCACUGCGCC---UUAAUUAAUC                   148
MIR164A1      GCCCGUCUUCUCCAUCGUGACCACUGCAACC-UUUGCAUAUCC                   142
MIR164A2      GCCCGUCUUCCCCAUCGUGACCACUGCAAAC-CUU--------                   132
```

**Supplementary Figure S5**: Loci encoding *miR164* in *C. pepo*. (A) Phylogenetic tree and (B) secondary structure of six *pri-miRNA164* encoded by different genes in *C. pepo*. Black arrows indicate the position of miR164 mature sequence. (C) Multiple alignment of the *MIR164* transcripts of *C. pepo*. Grey shadow shows the mature *miR164*.

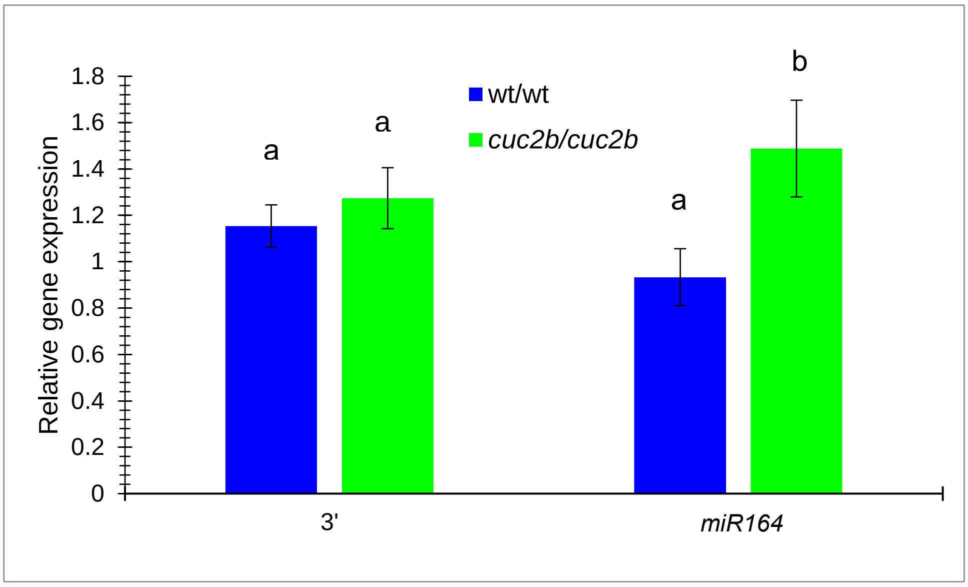


**Supplementary Figure S6**: qRT-PCR of *CpCUC2B* gene in the apical shoots of WT and *cuc2b* plants after female flowering. Left: Relative gene expression obtained with primers designed in the 3’ region of the gene. Right: Relative gene expression obtained with primers designed in the flanking regions of *miR164* binding site. Different letters indicate significant differences between samples for each pair of primers (ANOVA, $p \leq 0.05$); nd, no detectable expression.

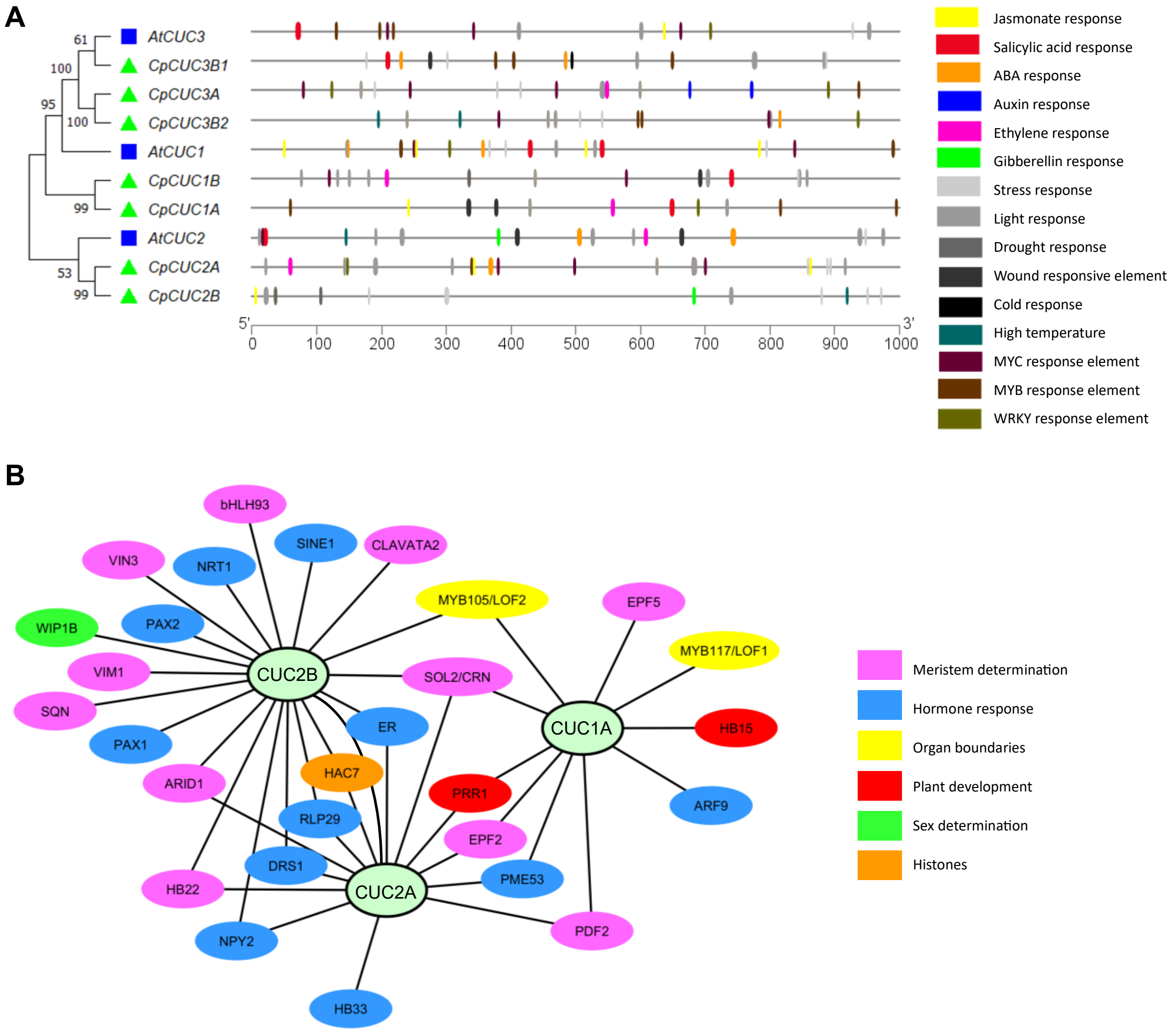


**Supplementary Figure S7.** *CUC* genes regulation network. **(A)** Cis-element analysis of *CUC* genes promoters. Elements related to hormones, stresses, and other transcription factors are shown. (**B**) Gene regulatory network of CUC transcription factors predicted by ARACNE algorithm.

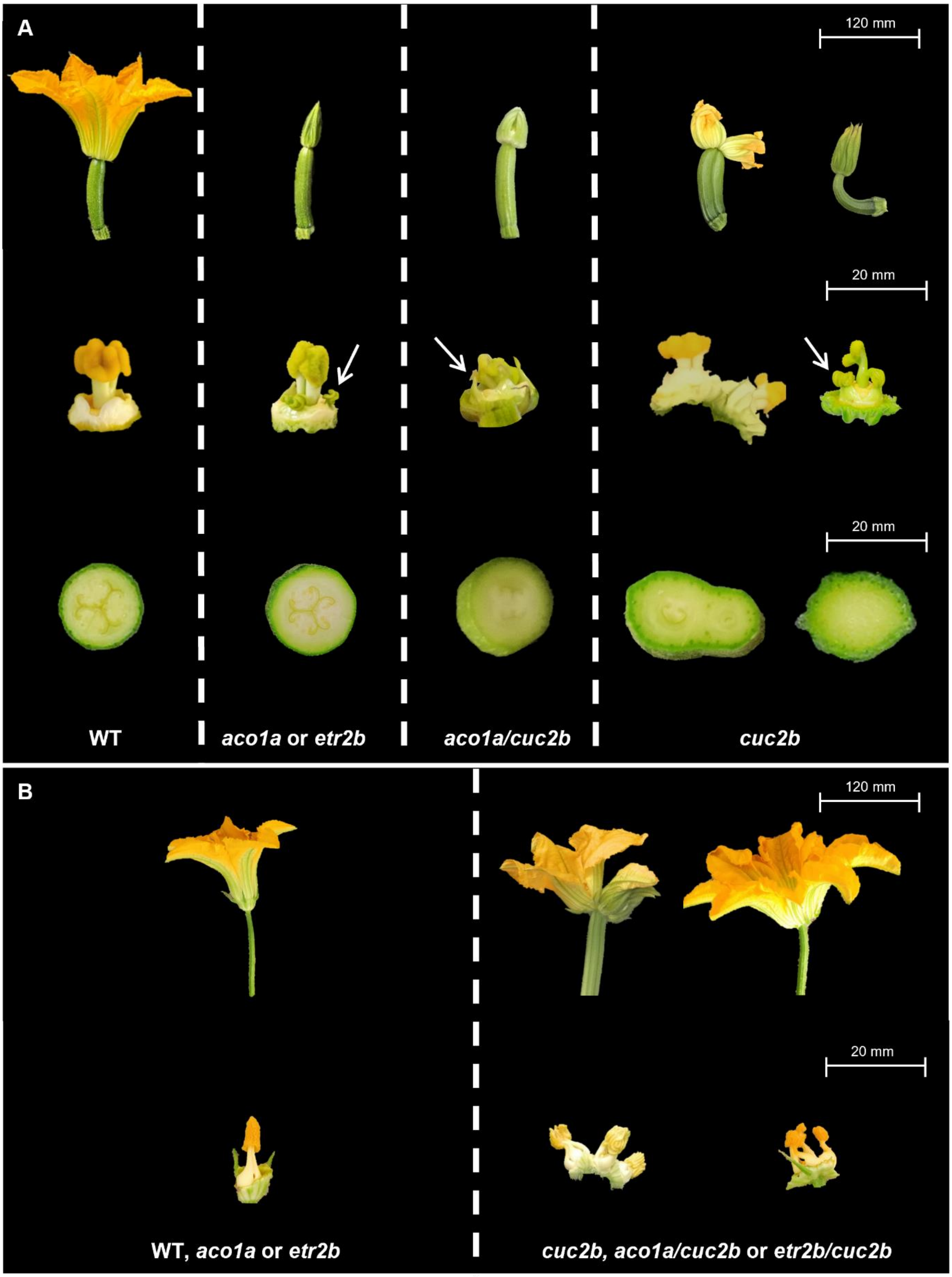


**Supplementary Figure S8.** Phenotypes of single and double mutants of *etr2b*, *aco1a* and *cuc2b*. **(A)** Pistillate flowers of each genotype. Up, complete pistillate flower; middle, detail of style and stigma of pistillate flowers; and down, transverse sections of ovaries. WT female flowers develop style and stigma, while bisexual *aco1a* and *etr2b* flowers develops both carpels and stamens. The double mutant *aco1a/cuc2b* produces bisexual flowers with stamens and defective carpels. Only 2.4% of the total flowers of *cuc2b* were bisexual, developing stamens and defective carpels, and an ovary with no endocarp and placental tissue. The double mutant *etr2b/cuc2b* only produces male flowers. White arrows indicate stamens of pistillate flowers. **(B)** Male flowers of the different genotypes. WT, *aco1a* and *etr2b* plants developed regular male flowers. The single *cuc2b* and the double *aco1a/cuc2b* and *etr2b/cuc2b* mutants develop single or double male flowers with increased number of floral organs.
